# m6A methylation regulators as predictors for treatment of advanced urothelial carcinoma with anti-PDL1 agent

**DOI:** 10.3389/fimmu.2022.1014861

**Published:** 2022-09-15

**Authors:** Jianqiu Kong, Sihong Lu, Long Zhang, Yuhui Yao, Jie Zhang, Zefeng Shen, Mingli Luo, Bin Liu, Junjiong Zheng, Tianxin Lin

**Affiliations:** ^1^ Department of Urology, Sun Yat-sen Memorial Hospital, Sun Yat-sen University, Guangzhou, China; ^2^ Department of Pathology, Sun Yat-sen Memorial Hospital, Sun Yat-sen University, Guangzhou, China; ^3^ Department of Internal Medicine, College of Medicine-Phoenix, University of Arizona, Phoenix, AZ, United States; ^4^ Guangdong Provincial Key Laboratory of Malignant Tumor Epigenetics and Gene Regulation, Sun Yat-sen Memorial Hospital, Sun Yat-sen University, Guangzhou, China; ^5^ Guangdong Provincial Clinical Research Center for Urological Diseases, Sun Yat-sen Memorial Hospital, Guangzhou, China

**Keywords:** m6A methylation regulators, urothelial carcinoma, PD1/PDL1, prediction, outcome

## Abstract

**Purpose:**

Immune checkpoint blockade agents were shown to provide a survival advantage in urothelial carcinoma, while some patients got minimal benefit or side effects. Therefore, we aimed to investigate the prognostic value of m6A methylation regulators, and developed a nomogram for predicting the response to atezolizumab in urothelial carcinoma patients.

**Methods:**

A total of 298 advanced urothelial carcinoma patients with response data in the IMvigor210 cohort were included. Differential expressions of 23 m6A methylation regulators in different treatment outcomes were conducted. Subsequently, a gene signature was developed in the training set using the least absolute shrinkage and selection operator (LASSO) regression. Based on the multivariable logistic regression, a nomogram was constructed by incorporating the gene signature and independent clinicopathological predictors. The performance of the nomogram was assessed by its discrimination, calibration, and clinical utility with internal validation.

**Results:**

Six m6A methylation regulators, including *IGF2BP1*, *IGF2BP3*, *YTHDF2*, *HNRNPA2B1*, *FMR1*, and *FTO*, were significantly differentially expressed between the responders and non-responders. These six regulators were also significantly correlated with the treatment outcomes. Based on the LASSO regression analysis, the gene signature consisting of two selected m6A methylation regulators (*FMR1* and *HNRNPA2B1*) was constructed and showed favorable discrimination. The nomogram integrating the gene signature, TMB, and PD-L1 expression on immune cells, showed favorable calibration and discrimination in the training set (AUC 0.768), which was confirmed in the validation set (AUC 0.755). Decision curve analysis confirmed the potential clinical usefulness of the nomogram.

**Conclusions:**

This study confirmed the prognostic value of *FMR1* and *HNRNPA2B1*, and constructed a nomogram for individualized prediction of the response to atezolizumab in patients with urothelial carcinoma, which may aid in making treatment strategies.

## Introduction

Urothelial carcinoma is one of the most common cancers worldwide (1), and the bladder is the usual site of occurrence (2). Due to the high recurrence rate and complicated therapeutic strategies, bladder cancer (BCa) is considered the most expensive tumor, which has brought a heavy economic burden to patients and society (3). Notably, a considerable proportion of urothelial carcinoma patients develop metastases during follow-up after radical therapies. The prognosis for advanced urothelial carcinoma remains poor (4). Emerging immunotherapy heralds a new era for the treatment of urothelial carcinoma. For the past few years, immunotherapy for malignant tumors has achieved many encouraging breakthroughs, making it the fourth treatment technique for cancer therapy after the operation, radiation therapy, and chemotherapy (5).

Currently, blockade of immune checkpoint molecule, programmed cell death 1 (PD1), or its ligand, PD ligand 1 (PDL1), was shown to provide a survival advantage in numbers of different advanced malignancies (6, 7). Effective as it is, only a subset of patients experienced durable responses and long-term survival after anti-PD1/PDL1 therapy, and the majority of patients achieved minimal or no clinical benefit (8). For example, the effective response rate for BCa is approximately 20% (9). Meanwhile, immunotherapy may cause adverse effects, and some may even lead to serious or life-threatening consequences (10, 11). Therefore, the optimization of individualized treatment has been listed as one of the top ten challenges of immunotherapy for tumors (12). How to identify the patients who are prone to have a good response to anti-PD1/PDL1 therapy is the current focus of intense research efforts. Many biomarkers have been reported to be predictive of cancer response to immunotherapy. The immunity system extends from systems-level principles of immune cell connectivity down to mechanistic characterization of individual receptors, which could provide potential opportunities for therapeutic intervention (13). Of these, tumor mutational burden (TMB) quantifying the number of somatic mutations in the tumor, CD8^+^ T-cell abundance, and PDL1 expression are commonly used predictors (9, 14, 15). However, their predictive efficacy may vary in specific cancer types (9, 12).

N6-methyladenosine (m6A) modification represents one of the most common chemical modifications in eukaryotic mRNA, which is a reversible process regulated by the balanced activities of methyltransferases, binding proteins, and demethylases, also known as “writers”, “readers” and “erasers” (16). Studies have demonstrated that m6A plays an important role in mRNA splicing, localization, translation, export, degradation, and stability (17–19). In addition, substantial evidence showed that dysregulated expression and genetic changes of m6A methylation regulators were associated with multiple biological disorders including dysregulated cell proliferation, differentiation and death, developmental defects, cancer progression, damaged self-renewal capacity, and aberrant immune regulation (20–22). Moreover, m6A methylation regulators also played critical roles in the development and progression of BCa by promoting cancer cell proliferation, self-renewal of cancer stem cells and so on (23–25). Besides, m6A regulators were reported to serve as reliable biomarkers to predict the treatment response and/or prognosis in BCa (26) as well as other tumors (27–29). Nonetheless, whether m6A regulators could aid in the prediction of immunotherapy response in urothelial carcinoma remains unknown.

In the present study, we systematically analyzed the association between the expression of 23 widely reported m6A regulators and the anti-PDL1 treatment (i.e., atezolizumab) response in advanced urothelial carcinoma patients. And we developed and validated a nomogram that integrated a gene signature derived from pre-treatment expression of m6A regulators and clinical variables for individualized prediction of the response to atezolizumab treatment in patients with urothelial carcinoma.

## Methods

### Data acquisition

Under the Creative Commons 3.0 license, standardized RNA-sequencing data and corresponding clinicopathological data, including TMB, PD-L1 expression on immune cells (IC), and tumor cells (TC), for the IMvigor210 cohort were extracted from the *IMvigor210CoreBiologies* R package (http://research-pub.gene.com/IMvigor210CoreBiologies/) developed by Mariathasan et al (30). Tumor specimens were scored *via* immunohistochemistry for PD-L1 expression on immune cells as IC0, IC1, IC2, or IC3 if <1%, ≥1% but <5%, ≥5% but <10%, or ≥10% of immune cells were PD-L1 positive, respectively. Besides, tumor tissue samples were scored as TC0, TC1, TC2, or TC3 if <1%, ≥1% but <5%, ≥5% but <50%, or ≥50% of tumor cells were PD-L1 positive, respectively. RNA-seq count data were transformed into Transcripts Per Million (TPM). Among 348 bladder cancer patients in the IMvigor210 cohort, we excluded those patients without treatment response data. Therefore, a total of 298 patients were finally included in our study (**Supplementary Table S1**). A reduction of tumor volume over 10% is defined as partial response (RECIST v1.1). All patients were classified into responders (complete and partial response) and non-responders (stable and progressive disease).

### Atezolizumab treatment response associated m6A methylation regulators

To explore the role of m6A methylation regulators in atezolizumab treatment, their differential expressions in different treatment outcomes were analyzed in all enrolled patients. The expressions of m6A methylation regulators were compared between the response group and non-response group using Wilcoxon’s test. To further understand the interactions among 23 m6A regulators, their expression correlations were evaluated using the correlation plot and the Spearman correlation test.

### Functional enrichment annotation

Metascape (http://metascape.org) is an online analysis tool designed to provide a comprehensive gene list annotation and analysis resource for experimental biologists, including gene annotation, functional enrichment, and construction of protein-protein interaction networks (31). In this study, we used Metascape to conduct the pathway and process enrichment of the m6A methylation regulators.

### Construction of the gene signature and evaluation of performance

The model construction flowchart of this study is presented in **Supplementary Figure S1**. All enrolled patients were randomly divided into two groups at a ratio of 7:3. As a result, 209 patients were allocated to the training set, whereas 89 patients were allocated to the independent validation set. In the training set, the univariable logistic regression analyses were used to measure the potential associations between 23 m6A regulators and the therapeutic outcomes. And the least absolute shrinkage and selection operator (LASSO) regression algorithm was performed to select treatment response-related genes with nonzero coefficients among 23 m6A regulators (32). An m6A-related gene signature was developed to evaluate the probability of treatment outcome for each patient using the gene score, which was calculated as a linear combination of the selected genes weighted by their respective coefficients. The discrimination of the gene signature was estimated by the area under the receiver operator characteristic (ROC) curve (AUC) in the training set and then validated in the validation set.

### Weighted gene co-expression network analysis

We used genes in the IMvigor210 dataset that were in the top 25% of variance from responders and non-responders to construct a weighted gene co-expression network analysis (WGCNA). Detailed descriptions regarding the WGCNA are shown in **Supplementary Methods**. To ensure the reliability of the WGCNA result, outlier samples that were distant from other samples were removed. An appropriate power cut-off threshold was selected to generate a scale-free topology overlap matric (TOM) and average linkage hierarchical clustering was used to detect gene modules. With the Dynamic Tree-Cut algorithm, gene modules were displayed as branched of dendrogram. The significance and correlation of module eigengenes of each gene module were generated. Then, we explored whether the module that most significantly correlated to treatment response contains m6A-related genes.

### Relationship of treatment outcome-related genes with immune infiltration patterns

The CIBERSORT algorithm was utilized to estimate the infiltration of 22 types of immune cells in all samples (33). Furthermore, to further investigate the role of treatment outcome-related genes in atezolizumab therapy, the relationship of those m6A methylation regulators selected in the LASSO regression analysis with different types of immune cells were analyzed.

### Construction of the nomogram

After univariable logistic regression analyses, the variables with *P* < 0.05 in the regression analyses were included in the following multivariable analysis in the training set. Backward stepwise selection using Akaike’s Information Criterion (AIC) was used to identify the significant predictors to develop the prediction model. A variance inflation factor (VIF) was calculated to assess the collinearity diagnostics of the multivariable logistic regression. According to the results of the multivariable logistic analysis, a nomogram was then constructed. A response score for each patient was calculated based on the multivariable logistic regression formula to reflect the probability of treatment response.

### Assessment of performance of the nomogram

In the training set, the AUC was used to measure the discrimination performance of the nomogram. In addition, a calibration curve was performed to estimate the calibration of the nomogram, along with the Hosmer-Lemeshow test to assess the goodness-of-fit (34).

### Validation of the nomogram

The performance of the nomogram was subsequently validated in the validation set. A response score can be calculated for each patient in the validation set by using the formula constructed in the training set. The AUC was then calculated, and the calibration curve and the Hosmer-Lemeshow test were conducted.

### Clinical usefulness of the nomogram

All patients were categorized into the predicted response or the predicted non-response groups according to their response scores, whose optimal cut-off point value was determined by the maximum Youden index in the training set (35). The log-rank test was performed to compare the Kaplan-Meier overall survival curves of the predicted response and the predicted non-response groups in the training and validation sets. Moreover, to determine the clinical usefulness of the nomogram, a decision curve analysis (DCA) was performed by calculating the net benefits for different threshold probabilities using the training and validation sets separately (36).

### Statistical analysis

All statistical tests were conducted using R statistical software (version 4.0.4; R Foundation for Statistical Computing). R packages used in this study, detailed descriptions regarding the LASSO algorithm, and DCA are available in **Supplementary Methods**. A two-sided *P*-value < 0.05 was considered statistically significant.

## Results

### Patient clinical characteristics

Patient clinical characteristics in the training and validation sets are shown in **Table 1**. Totally, 22.8% (68/298) of patients achieved complete response/partial response after atezolizumab treatment. In addition, 189 patients (63.4%) were dead during the follow-up. The median follow-up was 10.3 months (Interquartile range, 4.4–18.8). No significant difference was found between the training and validation set regarding the clinical characteristics (**Table 1**).

**Table 1:** Baseline characteristics of the patients.

Characteristic	Training set (n = 209)	Validation set (n = 89)	*P*
** Sex**			
Male	164 (78.5)	69 (77.5)	0.979
Female	45 (21.5)	20 (22.5)	
**IC**			
IC0	59 (28.2)	25 (28.1)	0.757
IC1	81 (38.8)	31 (34.8)	
IC2	69 (33.0)	33 (37.1)	
TC*			
TC0	164 (78.8)	74 (83.1)	0.494
TC1	14 (6.7)	3 (3.4)	
TC2	30 (14.4)	12 (13.5)	
**TMB, mut/Mb^†^**			
Median (Interquartile range)	8 [5, 14]	8 [5, 14]	0.662
**Treatment response**			
Complete response	14 (6.7)	11 (12.4)	0.059
Partial response	26 (12.4)	17 (19.1)	
Stable disease	42 (20.1)	21 (23.6)	
Progressive disease	127 (60.8)	40 (44.9)	
**Gene score**			
Median (Interquartile range)	-1.490 [-1.615, -1.352]	-1.475 [-1.597, -1.286]	0.355

Data are presented as No. (%) unless indicated otherwise.

P values were derived from the univariable association analyses between the training and validation set.

*One patient’s PD-L1 expression on tumor cells (TC) data was not available.

†TMB data were available for 161 and 73 patients in the training and validation sets, respectively.

### Atezolizumab treatment response associated m6A methylation regulators

**Figure 1:**
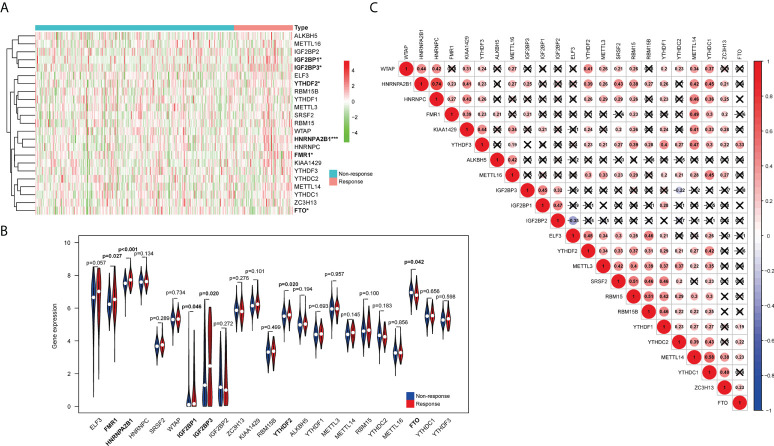
Relationship between the expression of m6A RNA methylation regulators and treatment response in urothelial carcinoma patients. **(A)** The heatmap shows the expression patterns of the 23 m6A methylation regulators between the response group and non-response group. **(B)** The violin plots exhibit the differential expression of the 23 m6A methylation regulators in the response group (red) and the non-response group (blue). **(C)** Spearman correlation analyses of the expression of the 23 m6A methylation regulators. *P < 0.05, ***P < 0.001.

**Figures 1A, B** show that six m6A methylation regulatory genes (*IGF2BP1*, *IGF2BP3*, *YTHDF2*, *HNRNPA2B1*, *FMR1*, and *FTO*) expressed differentially between the responders and non-responders. The expression levels of *IGF2BP1*, *IGF2BP3*, *YTHDF2*, *HNRNPA2B1*, and *FMR1* were significantly higher in the response group, while expression levels of *FTO* were significantly decreased in the non-response group. Among them, a significant difference in expression between bladder cancer and normal tissue in the TCGA-BLCA cohort is only detected in *IGF2BP3* (**Supplementary Figure S2**). The correlation heatmap indicated that *FMR1*, *YTHDF3*, *CBLL1*, *ZC3H13*, *METTL14*, *YTHDC1*, *KIAA1429*, and *LRPPRC* have a strong association with others (most r2>0.4; **Figure 1C**).

**Supplementary Figure S3** presents the results of the functional enrichment analysis obtained from Metascape. As shown in **Supplementary Figure S3A**, we found that several pathways were enriched, including regulation of mRNA metabolic process, regulation of mRNA stability, mRNA metabolic process, mRNA modification, regulation of mRNA process, mRNA transport, and negative regulation of mRNA metabolic process. The network of enriched terms can be found in **Supplementary Figure S3B** and **Table S2**. **Supplementary Figures S3C, D** presents the protein-protein interaction network and Molecular Complex Detection (MCODE) components. Five treatment response associated m6A regulators were found in the MCODE_1 component.

### Construction of the gene signature and evaluation of performance

**Figure 2:**
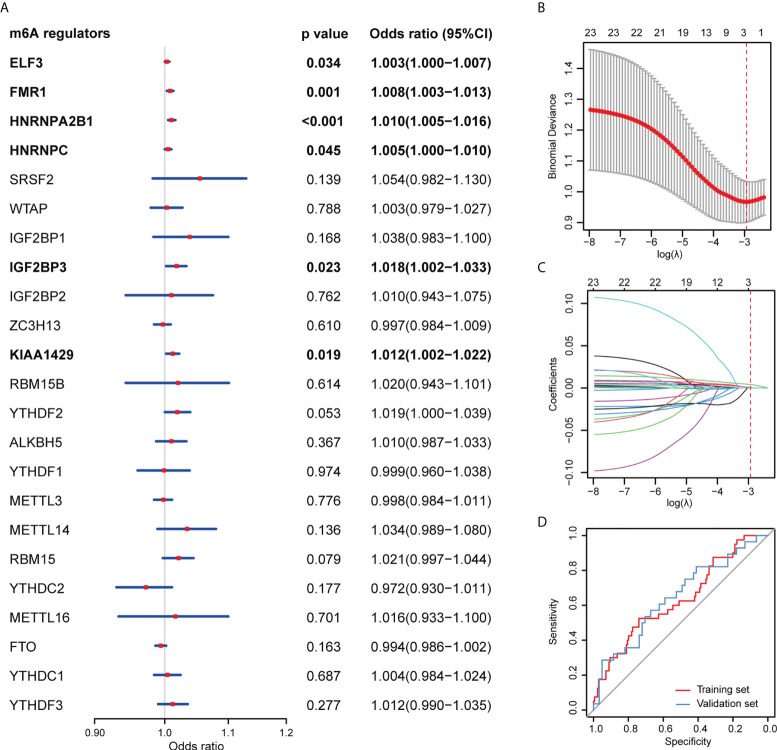
Construction and assessment of the m6A-related gene signature. **(A)** Univariable logistic regression analyses evaluating the predictive ability of m6A methylation regulators for treatment response of urothelial carcinoma patients. **(B)** Tuning parameter (λ) selection in the LASSO model used 10-fold cross-validation *via* minimum criteria. Binomial deviances from the LASSO regression cross-validation procedure were plotted as a function of log(λ). The numbers along the upper x-axis represent the average number of predictors. The red dots indicate the average deviance values for each model with a given λ, and the vertical bars through the red dots show the upper and lower values of the deviances. The dotted vertical lines are drawn at the optimal values where the model provides its best fit to the data. The optimal λ value of 0.053 with log (λ) = -2.936 was chosen. **(C)** LASSO coefficient profiles of the 23 m6A methylation regulators. The dotted vertical line is drawn at the value selected using 10-fold cross-validation in Figure 2B, where optimal λ resulted in 2 nonzero coefficients. **(D)** ROC curves of the gene signature in the training and validation sets.

In the univariable logistic regression analysis, *ELF3*, *FMR1*, *HNRNPA2B1*, *HNRNPC*, *IGF2BP3*, and *KIAA1429* were associated with the therapeutic outcomes in the training set (**Figure 2A**). Additionally, using the LASSO regression analysis, two treatment outcome-related genes (*FMR1* and *HNRNPA2B1*) with nonzero coefficients were selected in the training set (**Figures 2B, C**). Based on the LASSO logistic regression analysis, a gene signature was constructed, which can be calculated as a gene score for each patient: gene score = 0.000545 × *FMR1* expression level + 0.004127 × *HNRNPA2B1* expression level - 2.30373.

The gene signature showed favorable discrimination, with an AUC of 0.634 (95% confidence interval [CI] 0.535-0.733) in the training set, which was validated in the validation set with an AUC of 0.646 (95% CI 0.520-0.773; **Figure 2D**).

### Weighted gene co-expression network analysis

**Figure 3:**
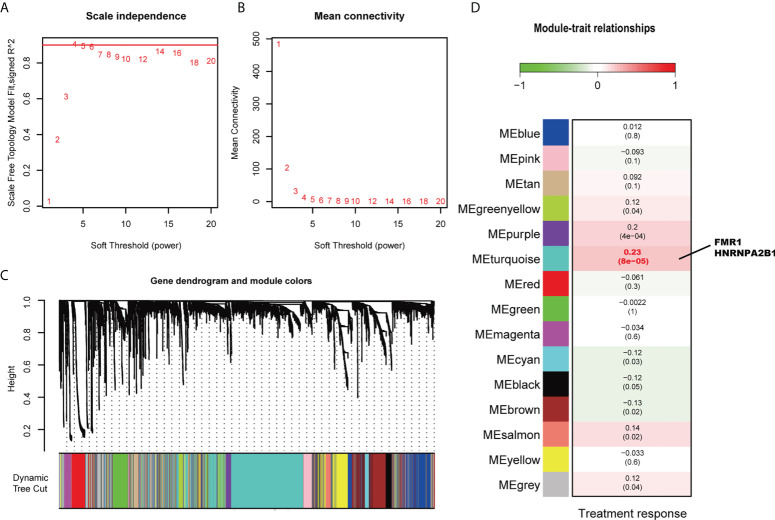
Weight Gene Co-expression Network Analysis. **(A)** Analysis of the scale-free index for various soft power thresholds. **(B)** Analysis of the mean connectivity of various soft power thresholds. **(C)** Dendrogram of the genes clustered based on a dissimilarity measure (1-TOM). **(D)** Average gene significances and errors in the modules associated with treatment response. The turquoise module was the most significantly correlated to treatment response. *FMR1* and *HNRNPA2B1* are in this module.

There was one outlier in the sample clustering (**Supplementary Figure S4**), which was excluded in the subsequent WGCNA. As 4 is the lowest value that allows obtaining more than 90% similarities in topology models (**Figures 3A, B**), a soft threshold power of 4 was selected. Finally, a total of 15 modules was obtained using a dynamic tree-cutting method (**Figure 3C**). Among these modules, the turquoise module was the most significantly correlated to treatment response (Pearson correlation coefficient = 0.23 and *P* < 0.001, **Figure 3D**). Of note, two identified treatment outcome-related genes, *FMR1* and *HNRNPA2B1*, are found in the turquoise module, indicating the important role of these two m6A regulators in the immunotherapy of bladder cancer.

Patients with low expression of *FMR1* and *HNRNPA2B1* were more likely to have death after receiving immunotherapy in the IMvigor210 cohort (**Supplementary Figure S5**). Their performance in prognostic prediction is also presented in **Supplementary Table S3**. However, we found that expression of *FMR1* and *HNRNPA2B1* were not correlated with the overall survival in bladder cancer patients based on TCGA-BLCA dataset, who were not treated with immunotherapy (**Supplementary Figure S6**). These results suggest that these two identified genes might influence the immunotherapy response through m6A methylation, affecting the prognosis of patients with urothelial carcinoma.

### Relationship of treatment outcome-related genes with immune infiltration patterns

**Figure 4:**
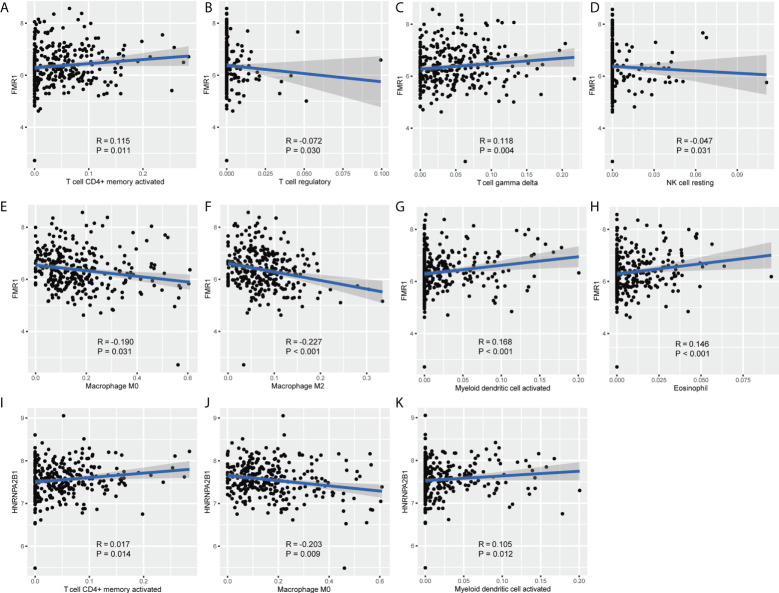
The relationship of *FMR1* and *HNRNPA2B1* with different types of immune cells. **(A–H)** Correlation plots show the relationship between *FMR1* and different types of immunocytes. **(I–K)** Correlation plots show the relationship between *HNRNPA2B1* and different types of immunocytes.

As shown in **Figure 4**, *FMR1* was negatively related to regulatory T cells, resting NK cells, M0 macrophages, M2 macrophages, was positively correlated with activated CD4+ memory T cells, gamma delta T cells, activated myeloid dendritic cells, and eosinophil. *HNRNPA2B1* was negatively related with M0 macrophages, and was positively correlated with activated CD4+ memory T cells and activated myeloid dendritic cells. Note that *FMR1* was most negatively correlated with M2 macrophages, and *HNRNPA2B1* was most negatively correlated with M0 macrophages.

### Construction of the nomogram and assessment of performance

**Figure 5:**
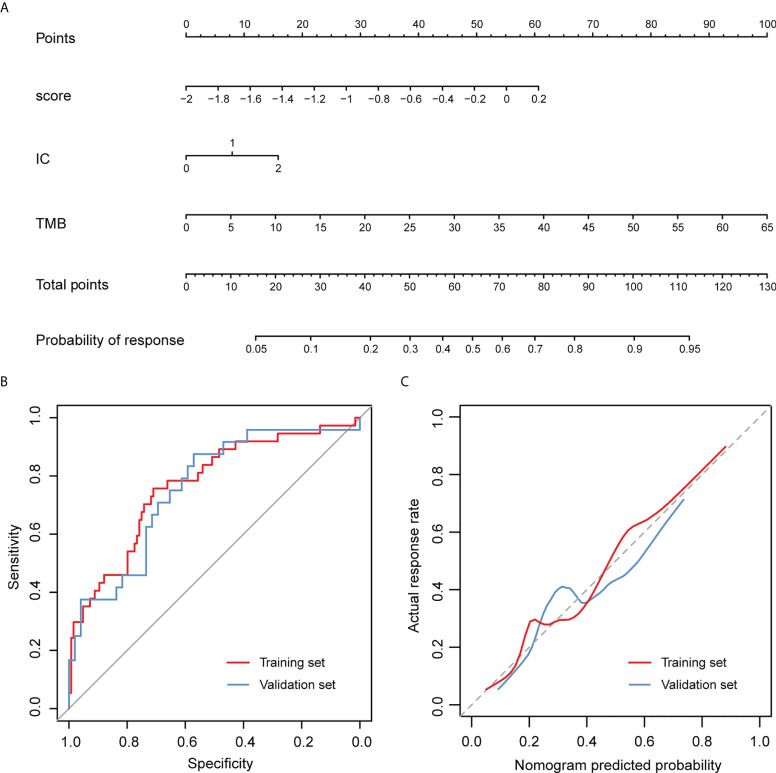
Nomogram to predict the response of atezolizumab treatment for patients with advanced urothelial carcinoma and its performance evaluation. **(A)** Points were assigned for gene score, IC and TMB by drawing a line upward from the corresponding values to the “Points” line. The sum of these three points, plotted on the “Total points” line, corresponds to predictions of the treatment response. **(B)** ROC curves of the nomogram. **(C)** Calibration curves of the nomogram. The observed treatment outcome is shown compared with the nomogram using the training set and validation set, respectively. The calibration curves depict the calibration of the nomogram in terms of the agreement between the predicted treatment outcomes and the observed treatment outcomes. The 45-degree dotted gray line represents a perfect prediction, and the solid lines represent the predictive performance of the nomogram. The distance between the solid line and the ideal line represents the superior predictive accuracy of the nomogram.

According to the univariate logistic regression analyses, three candidate variables were found to meet the threshold of *P* < 0.05, including the gene signature, IC, and TMB (**Table 2**). They were identified as the significant predictors of treatment outcomes in the subsequent multivariable logistic regression analysis. The VIF values ranged from 1.000 to 1.003, indicating that there was no collinearity in the collinearity diagnosis. By incorporating IC, TMB, and the gene signature, a nomogram was developed (**Figure 5A**) and the response score could be calculated for each patient to reflect the probability of treatment response based on the multivariable logistic regression formula. The calculating formula was as follow: response score = 1.673 × gene score + 0.481 × IC + 0.093 × TMB − 0.542. The predicted treatment response probability was calculated using 1/[1 + exp (−response score)].

**Table 2:** Univariate logistic regression analysis of the gene score and clinical candidate predictors in the training set.

Variables	Univariate logistic regression		Multivariate regression	
	OR (95% CI)	*P*	OR (95% CI)	*P*
**The gene score**	6.970 (1.567-35.815)	0.014^*^	5.330 (1.072-30.194)	0.044^*^
**Sex** (male vs. female)	2.171 (0.861-6.648)	0.130	–	–
**IC**	1.894 (1.129-3.316)	0.019^*^	1.618 (0.933-2.910)	0.095
**TC**	1.170 (0.723-1.815)	0.499	–	–
**TMB**	1.105 (1.055-1.168)	<0.001^*^	1.098 (1.047-1.163)	<0.001^*^

^*^P < 0.05.

CI, confidence interval; OR, odds ratio.

In the training set, an AUC of 0.768 (95% CI, 0.678-0.858) indicated that the nomogram had good discrimination (**Figure 5B**). The calibration curve of the nomogram estimating the probability of an effective treatment response demonstrated good agreement (**Figure 5C**), and the Hosmer-Lemeshow test yielded a non-significant statistic (*P* = 0.256), suggesting no departure from the perfect fit. The favorable calibration and discrimination performance of the nomogram was confirmed in the validation set, with an AUC of 0.755 (95% CI 0.636-0.875; **Figures 5B, C**). The Hosmer-Lemeshow test also demonstrated a non-significant statistic for the nomogram (*P* = 0.214).

### Clinical usefulness of the nomogram

**Figure 6:**
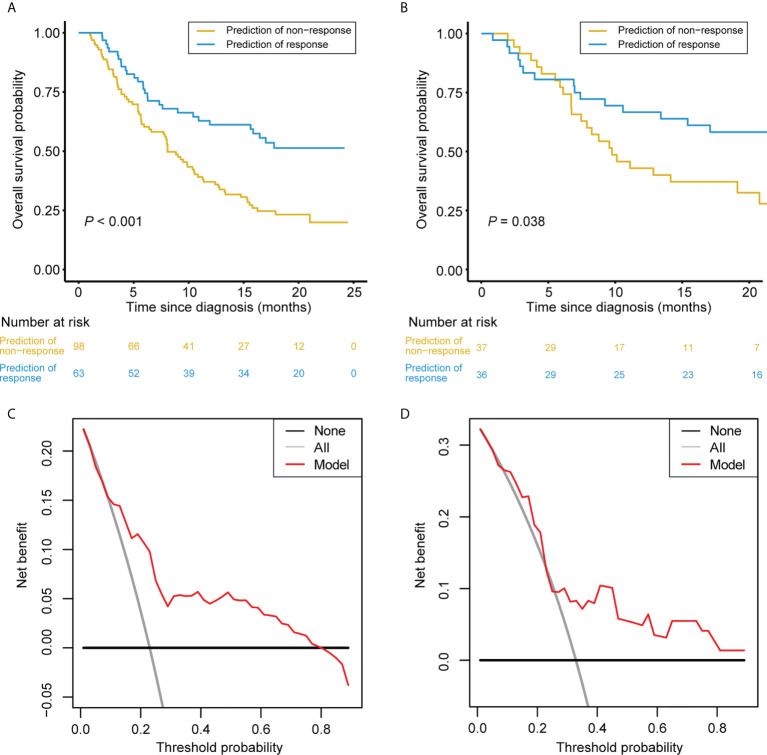
Clinical Usefulness of the Nomogram. **(A, B)** Kaplan-Meier survival curves of patients categorized into response and non-response groups in the training set **(A)** and validation set **(B)**, respectively. **(C, D)** DCA of the nomogram in the training set **(A)** and validation set **(B)**, respectively. The x-axis represents the threshold probability. The y-axis measures the net benefit. The black line depicts the net benefit of the strategy of treating no patients. The gray line depicts the net benefit of the strategy of treating all patients. The red line represents the nomogram. The net benefit was calculated by subtracting the proportion of all patients who are false positive from the proportion who are true positive, weighting by the relative harm of forgoing treatment compared with the negative consequences of unnecessary treatment. The threshold probability is where the expected benefit of treatment is equal to the expected benefit of avoiding treatment.

After obtaining the response scores from the nomogram, the patients were classified into the predicted response and the predicted non-response groups according to the optimal cutoff value of 0.194. Notably, in the training set, patients in the predicted response group had better OS compared with those in the predicted non-response group (**Figure 6A**); the same was true in the validation set (**Figure 6B**).

In the training and validation sets, the DCA suggested that using the nomogram to detect a treatment response adds more net benefit than either the treat-all or treat-none scheme for a wide range of threshold probability (**Figures 6C, D**).

## Discussion

Anti-PD1/PDL1 treatment has been increasingly recognized as a critical strategy in urothelial carcinoma. Precise targeting of patients is of great importance to increase benefits and cost-effectiveness. In this study, we determined the associations between m6A methylation regulators and atezolizumab treatment response. Furthermore, we developed a nomogram incorporating the m6A-related gene signature and clinical variables for individualized prediction of the response to atezolizumab in patients with urothelial carcinoma. This could aid in making treatment strategies and facilitate precision medicine.

In the study, differential expression analysis showed that six m6A methylation regulators, including *IGF2BP1*, *IGF2BP3*, *YTHDF2*, *HNRNPA2B1*, *FMR1*, and *FTO*, were significantly differentially expressed between the responders and non-responders. Moreover, the expression of these six regulators was significantly correlated with the treatment outcomes. These results may preliminarily indicate that these six m6A regulators have the potential of influencing the survival of urothelial carcinoma cells. Subsequently, we identified two critical m6A methylation regulators (i.e., *FMR1* and *HNRNPA2B1*) to develop an m6A-related gene signature for the prediction of the response to atezolizumab. The gene signature showed satisfactory discrimination with an AUC of 0.634 in the training set, which was further confirmed in the validation set with an AUC of 0.646.

Furthermore, after using multivariable logistic regression analysis to select candidate predictors, a nomogram was built by incorporating the gene signature, IC, and TMB. The nomogram demonstrated favorable calibration and discrimination in the training set (AUC 0.768) and also performed well in the validation set (AUC 0.755). Moreover, the DCA suggested that within a broad threshold probability, using the prediction tool to predict treatment response adds more benefit than the treat-all or the treat-none scheme. The presented nomogram could serve as a reliable prediction tool and inform a clinician how big the possibility is that a certain patient with advanced urothelial carcinoma would respond to atezolizumab treatment. Furthermore, this tool would aid in better risk stratification among these patients, which could allow better allocation of health resources and avoid adverse effects brought by atezolizumab on patients that would not respond well.

In our study, two treatment outcome-related m6A methylation regulators, i.e., *FMR1* and *HNRNPA2B1*, were determined by the LASSO regression analysis. And a high expression of *FMR1* and *HNRNPA2B1* indicated a favorable treatment outcome. The result of the prognostic value of *FMR1* is in line with previous research where the expression levels of *FMR1* were positively correlated with the overall survival of testicular germ cell tumors (37). On the other hand, the finding regarding *HNRNPA2B1* is contrary to other studies where high expression of *HNRNPA2B1* was significantly associated with poor prognosis in osteosarcoma (38), esophageal cancer (39) and adrenocortical carcinoma (40).

*FMR1 and HNRNPA2B1* were both regarded as m6A methylation reader (41–43). *FMR1* plays an important role in promoting m6A-modified mRNA nuclear export (44, 45) and interacts with m6A reader *YTHDF1* and *YTHDF2* to maintain the stability of its mRNA targets (43, 46, 47). To our knowledge, there is a lack of studies between *FMR1* and tumor immunity. In our study, *FMR1* was correlated with several types of tumor-infiltrating immune cells, suggesting that *FMR1* may be involved in the regulation of immune cells in the tumor microenvironment. *HNRNPA2B1* mediates mRNA slicing, primary microRNA processing and facilitates nucleocytoplasmic trafficking of mRNAs (41, 48–50). Previous studies have found that high expression of *HNRNPA2B1* promotes lymphatic metastasis (51)and recurrence (52)of bladder cancer. The function of *HNRNPA2B1* in tumor immunity remains controversial. Some studies have shown that *HNRNPA2B1* can promote tumor immunity and anti-tumor. For example, there is a significant positive correlation between *HNRNPA2B1* and M1 macrophages in esophageal cancer (39), and the expression of *HNRNPA2B1* is higher in M1 macrophages and T/NK cells than in other cells in glioblastoma (53). In contrast, other studies have revealed that *HNRNPA2B1* inhibits tumor immunity. For example, *HNRNPA2B1* is negatively correlated with the immune score, stromal score, and ESTIMATE in adrenal cortical cancer (40), as well as Th1 and Th17 in prostate cancer (54). In our study, *HNRNPA2B1* was positively correlated with activated CD4+ memory T cells and activated myeloid dendritic cells, implying that *HNRNPA2B1* may enhance the efficacy of immunotherapy through regulating the tumor-infiltrating immune cells. However, further experiments are needed to clarify the mechanism between these two genes and tumor immunity.

Of note, tumor mutation burden (TMB) has been found to be able to predict treatment efficacy of immune checkpoint blockade and has become a reliable biomarker for the identification of patients that will benefit from immunotherapy in many tumor types (55–57). In our study, patients with high TMB were prone to achieve a positive response. This is consistent with some previous studies which have shown that high TMB is associated with response to anti-CTLA-4 in melanoma (58, 59), and anti-PD1 in NSCLC (60). Given that high TMB is correlated with a greater likelihood of presenting cancer neoantigens on cancer cell surface (61), it is reasonable to speculate that those cancers with high TMB tend to respond to immune checkpoint blockade drugs as this greater mutation load may increase the probability of recognition by neoantigen-reactive T cells.

In addition, IC was positively correlated with an effective response in our study, which is in line with previous studies (62, 63). Webb et al. found that PD-L1 was mainly expressed by tumor-associated CD68^+^ macrophages rather than cancer cells, and showed a positive association with survival in high-grade serous carcinomas (62). PD-L1^+^ tumor-infiltrating lymphocytes densities were favorable prognostic indicators for progression-free (PFS) and overall survival (OS) (63).

Our study has several limitations. First, although m6A methylation regulatory genes have been found to have high prognostic values in the response to atezolizumab among advanced urothelial carcinoma patients, their specific mechanisms in urothelial carcinoma progression and prognosis are not yet clear and warranted to be further investigated by *in vitro* and *in vivo* experiments. Second, external validation in a larger dataset is needed to confirm the performance of the nomogram.

In summary, two critical m6A methylation regulators associated with immunotherapy in patients with advanced urothelial carcinoma were identified in our study. In addition, the presented nomogram derived from the m6A-related gene signature and clinical variables could serve as a reliable tool to predict the response to atezolizumab in advanced urothelial carcinoma. Further external validation is needed to determine the performance of the nomogram before its application in clinical practice.

## Data availability statement

The datasets presented in this study can be found in online repositories. The names of the repository/repositories and accession number(s) can be found in the article/**Supplementary Material**.

## Ethics statement

Ethical review and approval was not required for the study on human participants in accordance with the local legislation and institutional requirements. Written informed consent for participation was not required for this study in accordance with the national legislation and the institutional requirements.

## Author contributions

JK, SL, JJZ, and TL conceptualized and designed the study. LZ, YY, JZ, ZS, and ML did the literature research, performed the study selection, and data extraction. JK, JJZ, and BL analyzed and interpreted the data. BL, JJZ, and TL supervised the study. All the authors wrote, reviewed, and/or revised the manuscript. All authors contributed to the article and approved the submitted version.

## Funding

This study was supported by the China Postdoctoral Science Foundation (Grant No. 2021TQ0387, 2021M703709, 2022M713625), the National Natural Science Foundation of China (Grant No. 81825016), Project Supported by Guangdong Province Higher Vocational Colleges & Schools Pearl River Scholar Funded Scheme (for TL), Guangdong Provincial Clinical Research Center for Urological Diseases (2020B1111170006), the GuangDong Basic and Applied Basic Research Foundation (Grant No. 2020A1515111119).

## Conflict of interest

The authors declare that the research was conducted in the absence of any commercial or financial relationships that could be construed as a potential conflict of interest.

## Publisher’s note

All claims expressed in this article are solely those of the authors and do not necessarily represent those of their affiliated organizations, or those of the publisher, the editors and the reviewers. Any product that may be evaluated in this article, or claim that may be made by its manufacturer, is not guaranteed or endorsed by the publisher.

## Supplementary material

Supplementary Table 1 Data Source and Clinical Table.
