# Supplementary material for: m6A methylation regulators as predictors for treatment of advanced urothelial carcinoma with anti-PDL1 agent
Source: Front Immunol. 2022 Sep 15;13:1014861. doi: 10.3389/fimmu.2022.1014861 (PMC9521425; doi:10.3389/fimmu.2022.1014861)
Supplement: Supplementary file 1 [file DataSheet_1.doc]

###Figure 1

library("limma")

inputFile="m6Aexp.txt"

conNum=230

treatNum=68

outTab=data.frame()

grade=c(rep(1,conNum),rep(2,treatNum))

rt=read.table(inputFile,sep="\t",header=T,check.names=F)

rt=as.matrix(rt)

rownames(rt)=rt[,1]

exp=rt[,2:ncol(rt)]

dimnames=list(rownames(exp),colnames(exp))

data=matrix(as.numeric(as.matrix(exp)),nrow=nrow(exp),dimnames=dimnames)

data=avereps(data)

newGeneLists=c()

for(i in row.names(data)){

geneName=unlist(strsplit(i,"\\|",))[1]

geneName=gsub("\\/", "_", geneName)

rt=rbind(expression=data[i,],grade=grade)

rt=as.matrix(t(rt))

wilcoxTest<-wilcox.test(expression ~ grade, data=rt)

conGeneMeans=mean(data[i,1:conNum])

treatGeneMeans=mean(data[i,(conNum+1):ncol(data)])

logFC=log2(treatGeneMeans)-log2(conGeneMeans)

pvalue=wilcoxTest$p.value

conMed=median(data[i,1:conNum])

treatMed=median(data[i,(conNum+1):ncol(data)])

diffMed=treatMed-conMed

outTab=rbind(outTab,cbind(gene=i,conMean=conGeneMeans,treatMean=treatGeneMeans,logFC=logFC,pValue=pvalue))

if(pvalue<0.001){

newGeneLists=c(newGeneLists,paste0(i,"***"))

}else if(pvalue<0.01){

newGeneLists=c(newGeneLists,paste0(i,"**"))

}else if(pvalue<0.05){

newGeneLists=c(newGeneLists,paste0(i,"*"))

}else{

newGeneLists=c(newGeneLists,i)

}

}

write.table(outTab,file="diff.xls",sep="\t",row.names=F,quote=F)

heatmap=cbind(ID=newGeneLists,data)

write.table(heatmap,file="geneSigExp.txt",sep="\t",row.names=F,quote=F)

##Figure 1A

rt=read.table("geneSigExp.txt",sep="\t",header=T,row.names=1,check.names=F)

rt=log2(rt+1)

library(pheatmap)

Type=c(rep("nonresponse",230),rep("response",68))

names(Type)=colnames(rt)

Type=as.data.frame(Type)

pdf("heatmap.pdf",height=5,width=10)

pheatmap(rt,

annotation=Type,

color = colorRampPalette(c("green", "white", "red"))(50),

cluster_cols =F,

show_colnames = F,

scale="row",

fontsize = 10,

fontsize_row=10,

fontsize_col=3)

dev.off()

##Figure 1B

library(vioplot)

normal=230

tumor=68

rt=read.table("m6Aexp.txt",sep="\t",header=T,row.names=1,check.names=F)

rt=log2(rt+1)

rt=t(rt)

pdf("vioplot.pdf",height=6,width=12)

par(las=1,mar=c(4,5,3,3))

x=c(1:ncol(rt))

y=c(1:ncol(rt))

plot(x,y,

xlim=c(0,70),ylim=c(min(rt),max(rt)*1.1),

main="",xlab="", ylab="Gene expression",

pch=21,

cex.lab=1.5,

col="white",

xaxt="n")

for(i in 1:ncol(rt)){

normalData=rt[1:normal,i]

tumorData=rt[(normal+1):(normal+tumor),i]

vioplot(normalData,at=3*(i-1),lty=1,add = T,col = 'blue')

vioplot(tumorData,at=3*(i-1)+1,lty=1,add = T,col = 'red')

wilcoxTest=wilcox.test(normalData,tumorData)

p=round(wilcoxTest$p.value,3)

mx=max(c(normalData,tumorData))

lines(c(x=3*(i-1)+0.2,x=3*(i-1)+0.8),c(mx,mx))

text(x=3*(i-1)+0.5, y=mx*1.05, labels=ifelse(p<0.001, paste0("p<0.001"), paste0("p=",p)), cex = 0.8)

}

text(seq(1,70,3),0,xpd = NA,labels=colnames(rt),cex = 1,srt = 45,pos=2)

dev.off()

##Figure 1C

library(corrplot)

rt=read.table("m6Aexp.txt",sep="\t",header=T,row.names=1,check.names=F)

rt=t(rt)

res1 <- cor.mtest(rt, conf.level = 0.95)

pdf("correlation.pdf",height=16,width=16)

corrplot(corr=cor(rt),

method = "circle",

order = "hclust",

tl.col="black",

addCoef.col = "black",

p.mat = res1$p,

sig.level = 0.001,

insig = "pch",

number.cex = 1,

type = "upper",

col=colorRampPalette(c("blue", "white", "red"))(50),

)

dev.off()

###Figure 2

#logistic regression

d1<-read.table("m6Aexp&pathdata.csv",header=T,sep=",",row.names = 1)

covariates<-names(d1[,2:24])

univ_formulas <- sapply(covariates, function(x) as.formula(paste('response~', x)))

univ_formulas

univ_models<-lapply(univ_formulas,function(x){glm(x,data=d1,family=binomial())})

univ_models

univ_results<-lapply(univ_models,function(x){

p.value<-round(summary(x)$coefficients[2,4],digits=3)

beta<-round(coef(x)[2],digits=3);

OR<-round(as.matrix(exp(coef(x)))[2],digits=3);

OR.confint.lower<-round(as.matrix(exp(confint(x)))[2],3)

OR.confint.upper<-round(as.matrix(exp(confint(x)))[4],3)

OR<-paste0(OR," (",OR.confint.lower,"-",OR.confint.upper,")")

res<-c(beta,OR,p.value)

names(res)<-c("beta","OR (95% CI for OR)","p.value")

return(res)})

res<-t(as.data.frame(univ_results,check.names=FALSE))

table2<-as.data.frame(res)

write.csv(table2, file="uni logistic.csv")

##Figure 2A

library(forestplot)

options(forestplot_new_page = FALSE)

clrs <- fpColors(box="red",line="blue", summary="royalblue")

rt=read.table("uni logistic_forest.csv",header=T,sep=",",row.names=1,check.names=F) #table "uni logistic_forest.csv" was derived from table "uni logistic.csv"

data=as.matrix(rt)

HR=data[,1:3]

hr=sprintf("%.3f",HR[,"OR"])

hrLow=sprintf("%.3f",HR[,"OR.95L"])

hrHigh=sprintf("%.3f",HR[,"OR.95H"])

pVal=data[,"pvalue"]

pVal=ifelse(pVal<0.001, "<0.001", sprintf("%.3f", pVal))

tabletext <-

list(c(NA, rownames(HR)),

append("pvalue", pVal),

append("Odds ratio",paste0(hr,"(",hrLow,"-",hrHigh,")")) )

pdf(file="forest_logis.pdf",

width = 6,

height = 8,

)

forestplot(tabletext,

rbind(rep(NA, 3), HR),

col=clrs,

graph.pos=2,

graphwidth=unit(50, "mm"),

xlog=T,

lwd.ci=2,

boxsize=0.1,

xlab="Odds ratio",

xticks = c(0.9, 1.0,1.1, 1.2)

)

dev.off()

##Figure 2B&C

set.seed(666)

trainindex<-sample(1:nrow(d1),209)

trainset<-d1[trainindex,]

valset<-d1[-trainindex,]

trainset$group<-1

valset$group<-2

d1<-rbind(trainset,valset)

library(glmnet)

library(rms)

x<-as.matrix(trainset[c(2:24)])

y<-as.matrix(trainset[,1])

opar<-par(no.readonly=T)

par(pin=c(3,3))

set.seed(666)

cvfit = cv.glmnet(x, y, family = "binomial", type.measure = "deviance", alpha=1)

plot(cvfit)

cvfit$lambda.min

fit = glmnet(x, y, family = "binomial",alpha=1)

plot(fit, xvar = "lambda", label = F)

abline(v=log(cvfit$lambda.min),lty=2)

trainset$score<-predict(fit, newx = x, s = cvfit$lambda.min)

trainset<-transform(trainset,trainset$score)

xv<-as.matrix(valset[c(2:24)])

yv<-as.matrix(valset[,1])

valset$score<-predict(fit, newx = xv, s = cvfit$lambda.min)

valset<-transform(valset,valset$score)

all<-rbind(trainset,valset)

write.csv(all,file="m6Aexp&pathdata&score.csv")

##Figure 2D

library(pROC)

roc1<- roc(trainset$response,trainset$score)

auc(roc1)

ci.auc(roc1)

roc2<- roc(valset$response,valset$score)

auc(roc2)

ci.auc(roc2)

plot(roc1, col="red")

plot(roc2, col="blue",add=TRUE)

legend("bottomright",lty=1,lwd=2,bty="n",c("Training set","Validation set"),col = c("red","blue"))

###Figure 3

library(WGCNA)

options(stringsAsFactors = FALSE)

fpkm = read.table("IMvigor210_TPM_298.txt",sep="\t",header=T,check.names=F)

dim(fpkm)

names(fpkm)

datExpr0 = as.data.frame(t(fpkm[,-1]))

names(datExpr0) = fpkm$id;

rownames(datExpr0) = names(fpkm[,-1])

datExpr0

#check missing value

gsg = goodSamplesGenes(datExpr0, verbose = 3)

gsg$allOK #if TURE, then run the code

if (!gsg$allOK)

{

# Optionally, print the gene and sample names that were removed:

if (sum(!gsg$goodGenes)>0)

printFlush(paste("Removing genes:", paste(names(datExpr0)[!gsg$goodGenes], collapse = ", ")))

if (sum(!gsg$goodSamples)>0)

printFlush(paste("Removing samples:", paste(rownames(datExpr0)[!gsg$goodSamples], collapse = ", ")))

# Remove the offending genes and samples from the data:

datExpr0 = datExpr0[gsg$goodSamples, gsg$goodGenes]

}

#filter

meanFPKM=0.5

n=nrow(datExpr0)

datExpr0[n+1,]=apply(datExpr0[c(1:nrow(datExpr0)),],2,mean)

datExpr0=datExpr0[1:n,datExpr0[n+1,] > meanFPKM]

filtered_fpkm=t(datExpr0)

filtered_fpkm=data.frame(rownames(filtered_fpkm),filtered_fpkm)

names(filtered_fpkm)[1]="sample"

head(filtered_fpkm)

write.table(filtered_fpkm, file="FPKM_filter.xls",row.names=F, col.names=T,quote=FALSE,sep="\t")

expro= read.table("FPKM_filter.txt",sep="\t",header=T,check.names=F,row.names = 1)

m.vars=apply(expro,1,var)

expro.upper=expro[which(m.vars>quantile(m.vars, probs = seq(0, 1, 0.25))[4]),]

dim(expro.upper)

datExpr0=as.data.frame(t(expro.upper));

nGenes = ncol(datExpr0)

nSamples = nrow(datExpr0)

#Sample cluster

sampleTree = hclust(dist(datExpr0), method = "average")

par(cex = 0.6)

par(mar = c(0,4,2,0))

plot(sampleTree, main = "Sample clustering to detect outliers", sub="", xlab="", cex.lab = 1.5,

cex.axis = 1.5, cex.main = 2)

abline(h = 150000, col = "red")

dev.off()

#Determine cluster under the line

clust = cutreeStatic(sampleTree, cutHeight = 150000, minSize = 10)

table(clust)

#clust 1 contains the samples we want to keep.

keepSamples = (clust==1)

datExpr0 = datExpr0[keepSamples, ]

#Loading clinical trait data

traitData = read.table("response data.txt",row.names=1,header=T,check.names=F,sep="\t") #this table contains sample ID and treatment response data

dim(traitData)

names(traitData)

allTraits = traitData

dim(allTraits)

names(allTraits)

# Form a data frame analogous to expression data that will hold the clinical traits.

fpkmSamples = rownames(datExpr0)

traitSamples =rownames(allTraits)

traitRows = match(fpkmSamples, traitSamples)

datTraits = allTraits[traitRows,]

rownames(datTraits)

collectGarbage()

# Re-cluster samples

sampleTree2 = hclust(dist(datExpr0), method = "average")

# Convert traits to a color representation: white means low, red means high, grey means missing entry

traitColors = numbers2colors(datTraits, signed = FALSE)

pdf(file="2_Sample dendrogram and trait heatmap.pdf",width=12,height=12)

plotDendroAndColors(sampleTree2, traitColors,

groupLabels = names(datTraits),

main = "Sample dendrogram and trait heatmap")

dev.off()

#network construction

enableWGCNAThreads()

powers = c(c(1:10), seq(from = 12, to=20, by=2))

sft = pickSoftThreshold(datExpr0, powerVector = powers, verbose = 5)

##Figure 3A&B

pdf(file="3_Scale independence.pdf",width=9,height=5)

par(mfrow = c(1,2))

cex1 = 0.9

plot(sft$fitIndices[,1], -sign(sft$fitIndices[,3])*sft$fitIndices[,2],

xlab="Soft Threshold (power)",ylab="Scale Free Topology Model Fit,signed R^2",type="n",

main = paste("Scale independence"));

text(sft$fitIndices[,1], -sign(sft$fitIndices[,3])*sft$fitIndices[,2],

labels=powers,cex=cex1,col="red");

abline(h=0.90,col="red")

plot(sft$fitIndices[,1], sft$fitIndices[,5],

xlab="Soft Threshold (power)",ylab="Mean Connectivity", type="n",

main = paste("Mean connectivity"))

text(sft$fitIndices[,1], sft$fitIndices[,5], labels=powers, cex=cex1,col="red")

dev.off()

#chose the softPower

softPower =sft$powerEstimate

adjacency = adjacency(datExpr0, power = softPower)

# Turn adjacency into topological overlap

TOM = TOMsimilarity(adjacency);

dissTOM = 1-TOM

geneTree = hclust(as.dist(dissTOM), method = "average");

pdf(file="4_Gene clustering on TOM-based dissimilarity.pdf",width=12,height=9)

plot(geneTree, xlab="", sub="", main = "Gene clustering on TOM-based dissimilarity",

labels = FALSE, hang = 0.04)

dev.off()

minModuleSize = 30

dynamicMods = cutreeDynamic(dendro = geneTree, distM = dissTOM,

deepSplit = 2, pamRespectsDendro = FALSE,

minClusterSize = minModuleSize);

table(dynamicMods)

dynamicColors = labels2colors(dynamicMods)

table(dynamicColors)

##Figure 3C

pdf(file="5_Dynamic Tree Cut.pdf",width=8,height=6)

plotDendroAndColors(geneTree, dynamicColors, "Dynamic Tree Cut",

dendroLabels = FALSE, hang = 0.03,

addGuide = TRUE, guideHang = 0.05,

main = "Gene dendrogram and module colors")

dev.off()

# Calculate eigengenes

MEList = moduleEigengenes(datExpr0, colors = dynamicColors)

MEs = MEList$eigengenes

# Calculate dissimilarity of module eigengenes

MEDiss = 1-cor(MEs);

# Cluster module eigengenes

METree = hclust(as.dist(MEDiss), method = "average")

pdf(file="6_Clustering of module eigengenes.pdf",width=7,height=6)

plot(METree, main = "Clustering of module eigengenes",

xlab = "", sub = "")

MEDissThres = 0.25

abline(h=MEDissThres, col = "red")

dev.off()

# Call an automatic merging function

merge = mergeCloseModules(datExpr0, dynamicColors, cutHeight = MEDissThres, verbose = 3)

# The merged module colors

mergedColors = merge$colors

# Eigengenes of the new merged modules:

mergedMEs = merge$newMEs

pdf(file="7_merged dynamic.pdf", width = 9, height = 6)

plotDendroAndColors(geneTree, cbind(dynamicColors, mergedColors),

c("Dynamic Tree Cut", "Merged dynamic"),

dendroLabels = FALSE, hang = 0.03,

addGuide = TRUE, guideHang = 0.05)

dev.off()

# Rename to moduleColors

moduleColors = mergedColors

# Construct numerical labels corresponding to the colors

colorOrder = c("grey", standardColors(50))

moduleLabels = match(moduleColors, colorOrder)-1

MEs = mergedMEs

#relate modules to external clinical triats

nGenes = ncol(datExpr0)

nSamples = nrow(datExpr0)

moduleTraitCor = cor(MEs, datTraits, use = "p")

moduleTraitPvalue = corPvalueStudent(moduleTraitCor, nSamples)

##Figure 3D

pdf(file="8_Module-trait relationships.pdf",width=10,height=6)

textMatrix = paste(signif(moduleTraitCor, 2), "\n(",

signif(moduleTraitPvalue, 1), ")", sep = "")

dim(textMatrix) = dim(moduleTraitCor)

par(mar = c(6, 8.5, 3, 3))

labeledHeatmap(Matrix = moduleTraitCor,

xLabels = names(traitData),

yLabels = names(MEs),

ySymbols = names(MEs),

colorLabels = FALSE,

colors = greenWhiteRed(50),

textMatrix = textMatrix,

setStdMargins = FALSE,

cex.text = 0.5,

zlim = c(-1,1),

main = paste("Module-trait relationships"))

dev.off()

###Figure 4

d1<-read.table("m6Aexp_log&estimation_matrix.csv",header=T,row.names=1, sep=",")

library(ggplot2)

cor.test(d1$B.cell.naive_CIBERSORT,d1$FMR1,method="spearman")

cor.test(d1$B.cell.memory_CIBERSORT,d1$FMR1,method="spearman")

cor.test(d1$B.cell.plasma_CIBERSORT,d1$FMR1,method="spearman")

cor.test(d1$T.cell.CD8._CIBERSORT,d1$FMR1,method="spearman")

cor.test(d1$T.cell.CD4..naive_CIBERSORT,d1$FMR1,method="spearman")

cor.test(d1$T.cell.CD4..memory.resting_CIBERSORT,d1$FMR1,method="spearman")

cor.test(d1$T.cell.CD4..memory.activated_CIBERSORT,d1$FMR1,method="spearman") ####0.01074

cor.test(d1$T.cell.follicular.helper_CIBERSORT,d1$FMR1,method="spearman")

cor.test(d1$T.cell.regulatory..Tregs._CIBERSORT,d1$FMR1,method="spearman") ####0.02964

cor.test(d1$T.cell.gamma.delta_CIBERSORT,d1$FMR1,method="spearman") ####0.003977

cor.test(d1$NK.cell.resting_CIBERSORT,d1$FMR1,method="spearman") ####0.03089

cor.test(d1$NK.cell.activated_CIBERSORT,d1$FMR1,method="spearman")

cor.test(d1$Monocyte_CIBERSORT,d1$FMR1,method="spearman")

cor.test(d1$Macrophage.M0_CIBERSORT,d1$FMR1,method="spearman") ####0.03089

cor.test(d1$Macrophage.M1_CIBERSORT,d1$FMR1,method="spearman")

cor.test(d1$Macrophage.M2_CIBERSORT,d1$FMR1,method="spearman") ####0.0002273

cor.test(d1$Myeloid.dendritic.cell.resting_CIBERSORT,d1$FMR1,method="spearman")

cor.test(d1$Myeloid.dendritic.cell.activated_CIBERSORT,d1$FMR1,method="spearman") ####0.0004371

cor.test(d1$Mast.cell.activated_CIBERSORT,d1$FMR1,method="spearman")

cor.test(d1$Mast.cell.resting_CIBERSORT,d1$FMR1,method="spearman")

cor.test(d1$Eosinophil_CIBERSORT,d1$FMR1,method="spearman") ####0.0004371

cor.test(d1$Neutrophil_CIBERSORT,d1$FMR1,method="spearman")

cor(d1$T.cell.CD4..memory.activated_CIBERSORT,d1$FMR1)##0.11547

cor(d1$T.cell.regulatory..Tregs._CIBERSORT,d1$FMR1)##-0.0719058

cor(d1$T.cell.gamma.delta_CIBERSORT,d1$FMR1)##0.1179964

cor(d1$NK.cell.resting_CIBERSORT,d1$FMR1)## -0.04680992

cor(d1$Macrophage.M0_CIBERSORT,d1$FMR1)##-0.189758

cor(d1$Macrophage.M2_CIBERSORT,d1$FMR1)##-0.2270847

cor(d1$Myeloid.dendritic.cell.activated_CIBERSORT,d1$FMR1)##0.1678031

cor(d1$Eosinophil_CIBERSORT,d1$FMR1)##0.1457242

##Figure 4A-D

p1<-ggplot(d1,aes(T.cell.CD4..memory.activated_CIBERSORT,FMR1))+

geom_point()+

geom_smooth(method = "lm")

p2<-ggplot(d1,aes(T.cell.regulatory..Tregs._CIBERSORT,FMR1))+

geom_point()+

geom_smooth(method = "lm")

p3<-ggplot(d1,aes(T.cell.gamma.delta_CIBERSORT,FMR1))+

geom_point()+

geom_smooth(method = "lm")

p4<-ggplot(d1,aes(NK.cell.resting_CIBERSORT,FMR1))+

geom_point()+

geom_smooth(method = "lm")

ggarrange(p1, p2, p3, p4,labels = c("A", "B", "C","D"), ncol = 2, nrow = 2)

##Figure 4E-H

p1<-ggplot(d1,aes(Macrophage.M0_CIBERSORT,FMR1))+

geom_point()+

geom_smooth(method = "lm")

p2<-ggplot(d1,aes(Macrophage.M2_CIBERSORT,FMR1))+

geom_point()+

geom_smooth(method = "lm")

p3<-ggplot(d1,aes(Myeloid.dendritic.cell.activated_CIBERSORT,FMR1))+

geom_point()+

geom_smooth(method = "lm")

p4<-ggplot(d1,aes(Eosinophil_CIBERSORT,FMR1))+

geom_point()+

geom_smooth(method = "lm")

ggarrange(p1, p2, p3, p4,labels = c("E", "F", "G","H"), ncol = 2, nrow = 2)

cor.test(d1$B.cell.naive_CIBERSORT,d1$HNRNPA2B1,method="spearman")

cor.test(d1$B.cell.memory_CIBERSORT,d1$HNRNPA2B1,method="spearman")

cor.test(d1$B.cell.plasma_CIBERSORT,d1$HNRNPA2B1,method="spearman")

cor.test(d1$T.cell.CD8._CIBERSORT,d1$HNRNPA2B1,method="spearman")

cor.test(d1$T.cell.CD4..naive_CIBERSORT,d1$HNRNPA2B1,method="spearman")

cor.test(d1$T.cell.CD4..memory.resting_CIBERSORT,d1$HNRNPA2B1,method="spearman")

cor.test(d1$T.cell.CD4..memory.activated_CIBERSORT,d1$HNRNPA2B1,method="spearman") ####0.01406

cor.test(d1$T.cell.follicular.helper_CIBERSORT,d1$HNRNPA2B1,method="spearman")

cor.test(d1$T.cell.regulatory..Tregs._CIBERSORT,d1$HNRNPA2B1,method="spearman")

cor.test(d1$T.cell.gamma.delta_CIBERSORT,d1$HNRNPA2B1,method="spearman")

cor.test(d1$NK.cell.resting_CIBERSORT,d1$HNRNPA2B1,method="spearman")

cor.test(d1$NK.cell.activated_CIBERSORT,d1$HNRNPA2B1,method="spearman")

cor.test(d1$Monocyte_CIBERSORT,d1$HNRNPA2B1,method="spearman")

cor.test(d1$Macrophage.M0_CIBERSORT,d1$HNRNPA2B1,method="spearman")####0.009381

cor.test(d1$Macrophage.M1_CIBERSORT,d1$HNRNPA2B1,method="spearman")

cor.test(d1$Macrophage.M2_CIBERSORT,d1$HNRNPA2B1,method="spearman")

cor.test(d1$Myeloid.dendritic.cell.resting_CIBERSORT,d1$HNRNPA2B1,method="spearman")

cor.test(d1$Myeloid.dendritic.cell.activated_CIBERSORT,d1$HNRNPA2B1,method="spearman") #### 0.01235

cor.test(d1$Mast.cell.activated_CIBERSORT,d1$HNRNPA2B1,method="spearman")

cor.test(d1$Mast.cell.resting_CIBERSORT,d1$HNRNPA2B1,method="spearman")

cor.test(d1$Eosinophil_CIBERSORT,d1$HNRNPA2B1,method="spearman")

cor.test(d1$Neutrophil_CIBERSORT,d1$HNRNPA2B1,method="spearman")

cor(d1$T.cell.CD4..memory.activated_CIBERSORT,d1$HNRNPA2B1)## 0.1446121

cor(d1$Macrophage.M0_CIBERSORT,d1$HNRNPA2B1)## -0.2033304

cor(d1$Myeloid.dendritic.cell.activated_CIBERSORT,d1$HNRNPA2B1)##0.1054626

##Figure 4I-K

p1<-ggplot(d1,aes(T.cell.CD4..memory.activated_CIBERSORT,HNRNPA2B1))+

geom_point()+

geom_smooth(method = "lm")

p2<-ggplot(d1,aes(Macrophage.M0_CIBERSORT,HNRNPA2B1))+

geom_point()+

geom_smooth(method = "lm")

p3<-ggplot(d1,aes(Myeloid.dendritic.cell.activated_CIBERSORT,HNRNPA2B1))+

geom_point()+

geom_smooth(method = "lm")

ggarrange(p1, p2, p3,labels = c("I", "J", "K"), ncol = 2, nrow = 2)

###Figure 5

##model construction

d1<-read.table("m6Aexp&pathdata&score.csv",sep=",",header=T,row.names = 1)

d1<-subset(d1,TMB>=0)

trainset<-subset(d1,group==1)

valset<-subset(d1,group==2)

#Univariate logistic regression

f1<-glm(response ~ Sex,data=trainset,family=binomial())

summary(f1)

coef(f1)

exp(coef(f1))

exp(confint(f1))

f1<-glm(response ~ IC,data=trainset,family=binomial())

summary(f1)

coef(f1)

exp(coef(f1))

exp(confint(f1))

f1<-glm(response ~ TC,data=trainset,family=binomial())

summary(f1)

coef(f1)

exp(coef(f1))

exp(confint(f1))

f1<-glm(response ~ TMB,data=trainset,family=binomial())

summary(f1)

coef(f1)

exp(coef(f1))

exp(confint(f1))

f1<-glm(response ~ score,data=trainset,family=binomial())

summary(f1)

coef(f1)

exp(coef(f1))

exp(confint(f1))

#Multivariate regression

library(car)

fvif<-glm(response~score+IC+TMB,data=trainset,family=binomial())

vif(fvif) #collinearity diagnostics

library(MASS)

f1<-glm(response~score+IC+TMB,data=trainset,family=binomial())

stepAIC(f1,direction = "backward")

#final model

f1<-glm(response~score+IC+TMB,data=trainset,family=binomial())

summary(f1)

coef(f1)

exp(coef(f1))

exp(confint(f1))

d1$pre_nomo <- predict(f1,newdata=d1,type='response')

##Figure 5A

library(rms)

ddist<-datadist(trainset)

options(datadist="ddist")

f1<-lrm(response~score +IC+TMB,data = trainset)

nom1<-nomogram(f1,fun = plogis,fun.at = c(seq(.1,.9, by=.1),0.95,0.99,0.01,0.05),lp=F,funlabel = "Probability of response")

plot(nom1)

##Figure 5B

f1<-glm(response~score +IC+TMB,data = trainset,family=binomial())

pre1 <- predict(f1,type='response')

roc1<- roc(trainset$response,pre1)

auc(roc1)

ci.auc(roc1)

f2<-glm(valset$response~predict(f1,newdata = valset))

pre2 <- predict(f2,type='response')

roc2<- roc(valset$response,pre2)

auc(roc2)

ci.auc(roc2)

plot(roc1, col="red")

plot.roc(roc2, add=TRUE, col="blue")

legend("bottomright",lty=1,lwd=2,bty="n",c("Training set","Validation set"),col = c("red","blue"))

##Figure 5C

#training set

fc<-lrm(response~score +IC+TMB,data = trainset,x=T,y=T)

cal1<-calibrate(fc,group=trainset$response)

opar<-par(no.readonly=T)

par(pin=c(3,3))

plot(cal1,xlim = c(0,1.0),ylim = c(0,1.0))

par(opar)

#validation set

fv<-lrm(response~predict(fc,newdata=valset),x=T,y=T,data = valset)

calv<-calibrate(fv,group=valset$response)

opar<-par(no.readonly=T)

par(pin=c(3,3))

plot(calv)

plot(calv,xlim = c(0,1.0),ylim = c(0,1.0))

###Figure 6

##Figure 6A&B

library("survival")

library("survminer")

fit1 <- survfit(Surv(time, status) ~ subgroup, data = trainset)

ggsurvplot(fit1,

pval = TRUE,

conf.int = F,

censor=F,

risk.table = TRUE,

risk.table.col = "strata",

palette = c("#E7B800", "#2E9FDF"),

legend.labs = c("Prediction of non-response","Prediction of response"))

fit2 <- survfit(Surv(time, status) ~ subgroup, data = valset)

ggsurvplot(fit2,

pval = TRUE,

conf.int = F,

censor=F,

risk.table = TRUE,

risk.table.col = "strata",

palette = c("#E7B800", "#2E9FDF"),

legend.labs = c("Prediction of non-response","Prediction of response"),

xlim=c(0,20),

break.time.by=5)

##Figure 6C&D

source("dca.R")

#training set

f1<-glm(response~score +IC+TMB, data = trainset, family=binomial(link="logit"))

trainset$pred= predict(f1, type="response")

dca(data=trainset, outcome="response", predictors="pred",xstart=0.01, xstop=0.90, xby=0.02, ymin=-0.05, probability=T,smooth = T)

#validation set

valset$pred= predict(f1,newdata = valset, type="response")

dca(data=valset, outcome="response", predictors="pred",xstart=0.01, xstop=0.90, xby=0.02, ymin=-0.05, probability=T,smooth = T)
