# Supplementary material for: m6A methylation regulators as predictors for treatment of advanced urothelial carcinoma with anti-PDL1 agent
Source: Front Immunol. 2022 Sep 15;13:1014861. doi: 10.3389/fimmu.2022.1014861 (PMC9521425; doi:10.3389/fimmu.2022.1014861)
Supplement: Supplementary file 2 [file DataSheet_2.docx]

**Supplementary Material**

[Supplementary Methods 2](#_Toc4813)

[*S1. R packages used in this Study* 2](#_Toc11103)

[*S2. Weighted gene co-expression network analysis* 2](#_Toc29098)

[*S3. Detailed description of the LASSO method* 3](#_Toc6324)

[*S4. Decision curve analysis* 3](#_Toc30726)

[*S5. Youden Index* 4](#_Toc22064)

[SUPPLEMENTARY TABLES 6](#_Toc6911)

[Table S2. Top 9 clusters with their representative enriched terms. 6](#_Toc29598)

[Table S3. The value on the prognostic prediction of FMR1, HNRNPA2B1 and the combination of these two genes. 7](#_Toc26063)

[SUPPLEMENTARY FIGURES 8](#_Toc1915)

[Figure S1. The model construction flowchart. 8](#_Toc13421)

[Figure S2. IMvigor210 Pivotal Study for Atezolizumab. 9](#_Toc18349)

[Figure S3. Differentially expressed genes identified in the TCGA-BLCA cohort. 10](#_Toc4058)

[Figure S4. The function network of 23 m6A related genes. 11](#_Toc251)

[Figure S5. Sample clustering to detect outliers. 12](#_Toc8781)

[Figure S6. Kaplan–Meier survival analysis for the two selected m6A related genes in the IMvigor210 cohort. 13](#_Toc11045)

[Figure S7. Kaplan–Meier survival analysis for the two selected m6A related genes in the TCGA-BLCA cohort. 14](#_Toc10594)

# Supplementary Methods

## *S1. R packages used in this Study*

All statistical analyses were performed using R statistical software version 4.0.4 (<https://www.r-project.org/>). R packages used in this study are listed as follow:

| **Statistical analysis** | **R package** |
| --- | --- |
| Differential expression analyses | limma |
| Heatmap | pheatmap |
| Correlation plot | corrplot |
| Forest plot | forestplot |
| Weighted gene co-expression network analysis | WGCNA |
| LASSO regression analyses | glmnet |
| ROC curves | pROC |
| VIF values calculation | car |
| Calibration plot | rms |
| Hosmer-Lemeshow test | vcdExtra |
| Kaplan-Meier curve | Survival  survminer |
| Decision curve analysis | dca.R |

## *S2. Weighted gene co-expression network analysis*

Weighted gene co-expression network analysis (WGCNA) is a useful bioinformatics analyzing method by reconstructing gene co-expression modules and summarizing modules via module eigengenes (ME) and intramodular hub genes [[1](#_ENREF_1)]. It can be used to explore the module (cluster) structure in a network, to measure the relationships between genes and modules (module membership information), to explore the relationships among modules (eigengene networks), to rank-order genes or modules (e.g. with regard to their relationship with a sample trait), and to generate testable hypotheses for validation in independent data sets [[1](#_ENREF_1)]. This method is frequently used to explore the complex relationships between genes and phenotypes in oncology.

## *S3. Detailed description of the LASSO method*

LASSO is a powerful method for regression with high-dimensional predictors. In our study, the LASSO method was combined with a logistic regression model for analysis of the treatment response, which could select the most important m6A related genes from the training set. This method minimizes a log partial likelihood subject to the sum of the absolute values of the parameters being bounded by a constant:

$\hat{\beta}=argmin \mathcal{l}\left( \beta\right)$, subject to $\sum\left| \beta_{j} \right|\leq s$

where, $\hat{\beta}$ is the obtained parameters, $\mathcal{l}\left( \beta\right)$ is the log partial likelihood of the logistic regression model, $s＞0$ is a constant.

The LASSO method can be used for feature reduction and selection by shrinking coefficients and forcing certain coefficients to be set to zero through absolute constraint [[2](#_ENREF_2)]. In this study, the standardized constraint parameter $s$ was set as 0.053 and two nonzero coefficients ($\hat{\beta}$) were selected by LASSO.

## *S4. Decision curve analysis*

In the study, the decision curve analysis (DCA) method was used to estimate the clinical usefulness of the presented model. The DCA algorithm evaluates prediction models by calculating the range of threshold probabilities in which a prediction or prognostic model is clinically useful. DCA is a comprehensive method for assessing and comparing different diagnostic and prognostic models. The theory of DCA can be illustrated by the equation below:

$$\frac{a-c}{d-b}=\frac{1-P_{t}}{P_{t}}$$

where d – b represents the influence of unnecessary treatment. If treatment is directed by a prediction model, d – b is the harm related to a false-positive result compared with a true-negative result. Inversely, a – c represents the consequence of rejecting beneficial treatment, in other words, the harm from a false-negative result compared with a true-positive result. Pt represents where the expected benefit of treatment is equal to the expected benefit of refraining from treatment [[3](#_ENREF_3), [4](#_ENREF_4)].

## *S5. Youden Index*

Youden's index is a single statistic that captures the performance of a dichotomous diagnostic test. Informedness is its generalization to the multiclass case and estimates the probability of an informed decision. The index gives equal weight to false positive and false negative values, so all tests with the same value of the index give the same proportion of total misclassified results. While it is technically possible to obtain a value of less than zero from this equation, e.g. Classification yields only False Positives and False Negatives, a value of less than zero just indicates that the positive and negative labels have been switched. After correcting the labels the result will then be in the 0 through 1 range [5].

Youden index = Sensitivity + Specificity -1

Youden's index is often used in conjunction with receiver operating characteristic (ROC) analysis. The index is defined for all points of an ROC curve, and the maximum value of the index may be used as a criterion for selecting the optimum cut-off point when a diagnostic test gives a numeric rather than a dichotomous result. The index is represented graphically as the height above the chance line, and it is also equivalent to the area under the curve subtended by a single operating point [6].

**References**

1. Langfelder P, Horvath S. WGCNA: an R package for weighted correlation network analysis. BMC Bioinformatics 2008; 9: 559.

2. Tibshirani R. Regression shrinkage and selection via the lasso: a retrospective. J R Stat Soc Series B Stat Methodol 2011; 73: 273-282.

3. Balachandran VP, Gonen M, Smith JJ, DeMatteo RP. Nomograms in oncology: more than meets the eye. Lancet Oncol 2015; 16: e173-180.

4. Vickers AJ, Elkin EB. Decision curve analysis: a novel method for evaluating prediction models. Med Decis Making 2006; 26: 565-574.

5. YOUDEN W J,Index for rating diagnostic tests.[J] .Cancer, 1950, 3: 32-5.

6. Schisterman Enrique F,Perkins Neil J,Liu Aiyi et al. Optimal cut-point and its corresponding Youden Index to discriminate individuals using pooled blood samples. [J] . Epidemiology, 2005, 16: 73-81.

# SUPPLEMENTARY TABLES

## Table S2. Top 9 clusters with their representative enriched terms.

| **GO** | **Category** | **Description** | **Count** | **Log10(P)** | **Log10(q)** |
| --- | --- | --- | --- | --- | --- |
| GO:1903311 | GO Biological Processes | regulation of mRNA metabolic process | 20 | -36.95 | -32.6 |
| GO:0043488 | GO Biological Processes | regulation of mRNA stability | 13 | -23.66 | -19.61 |
| GO:0016071 | GO Biological Processes | mRNA metabolic process | 14 | -17.88 | -14.23 |
| GO:0009451 | GO Biological Processes | RNA modification | 10 | -16.4 | -13.01 |
| GO:0050684 | GO Biological Processes | regulation of mRNA processing | 9 | -15.17 | -11.87 |
| GO:0051028 | GO Biological Processes | mRNA transport | 8 | -13.38 | -10.29 |
| GO:1903312 | GO Biological Processes | negative regulation of mRNA metabolic process | 6 | -10.13 | -7.34 |
| CORUM:351 | CORUM | Spliceosome | 5 | -7.19 | -4.56 |
| GO:0034470 | GO Biological Processes | ncRNA processing | 3 | -2.47 | -0.13 |

"Count" is the number of genes. "Log10(P)" is the p-value in log base 10. "Log10(q)" is the multi-test adjusted p-value in log base 10.

## Table S3. The value on the prognostic prediction of FMR1, HNRNPA2B1 and the combination of these two genes.

| **Variate** | **C-index (95% CI)** |
| --- | --- |
| FMR1 | 0.554 (0.503-0.605) |
| HNRNPA2B1 | 0.519 (0.471-0.567) |
| FMR1 + HNRNPA2B1 | 0.547 (0.497-0.597) |

# SUPPLEMENTARY FIGURES


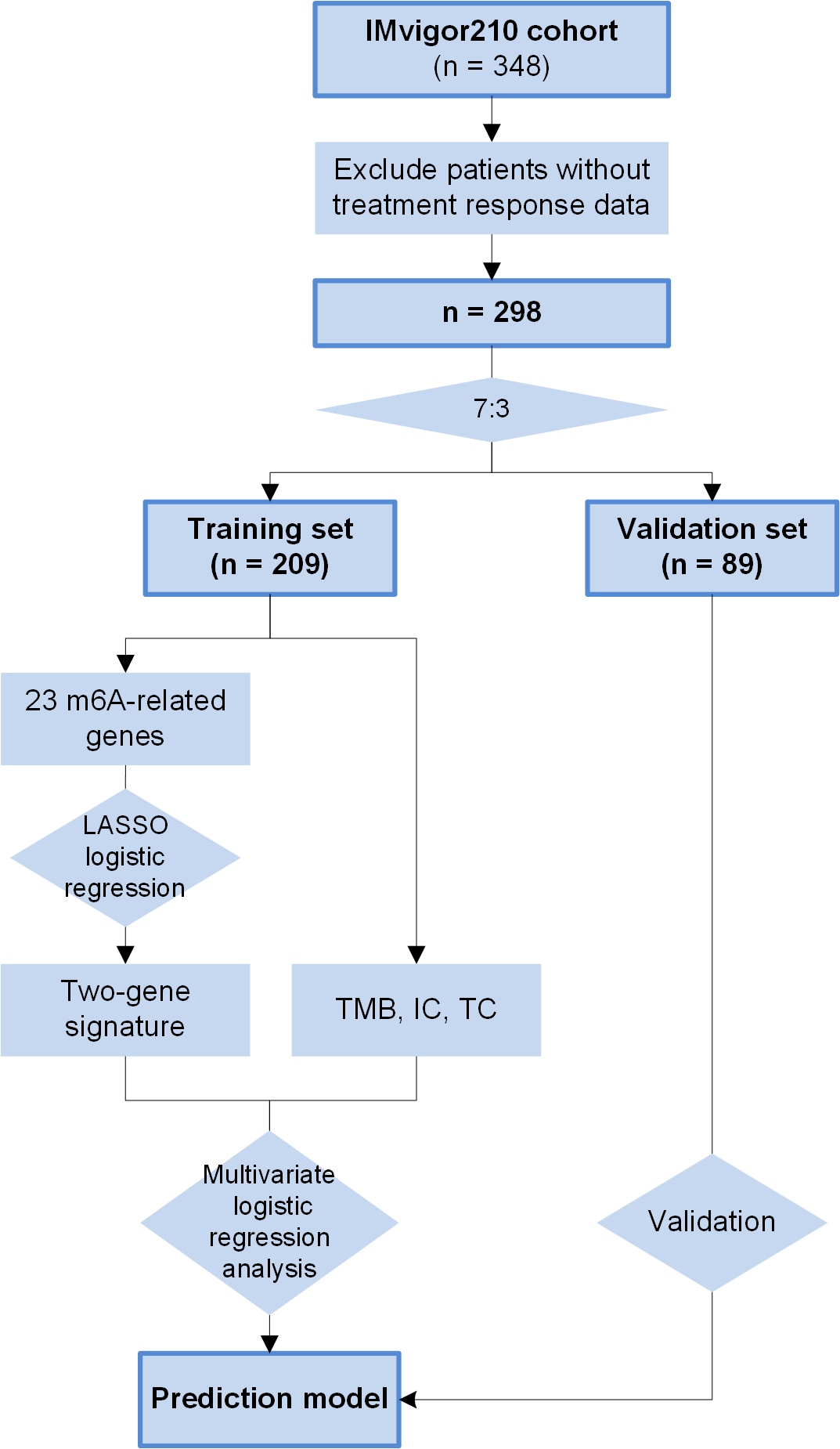


## Figure S1. The model construction flowchart.


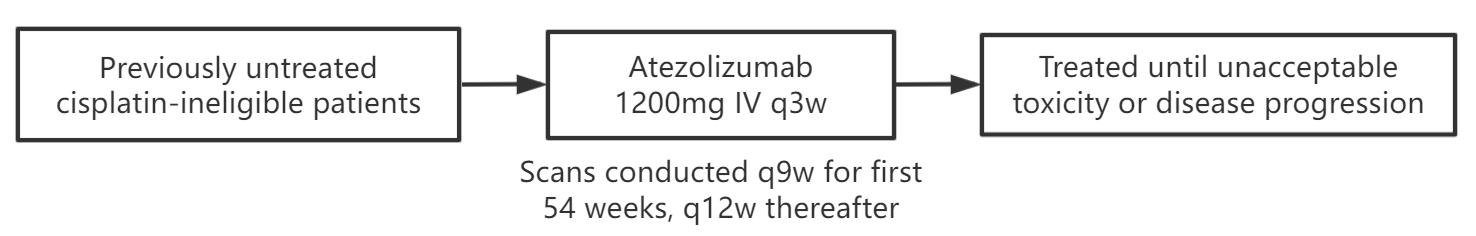


## Figure S2. IMvigor210 Pivotal Study for Atezolizumab.

**
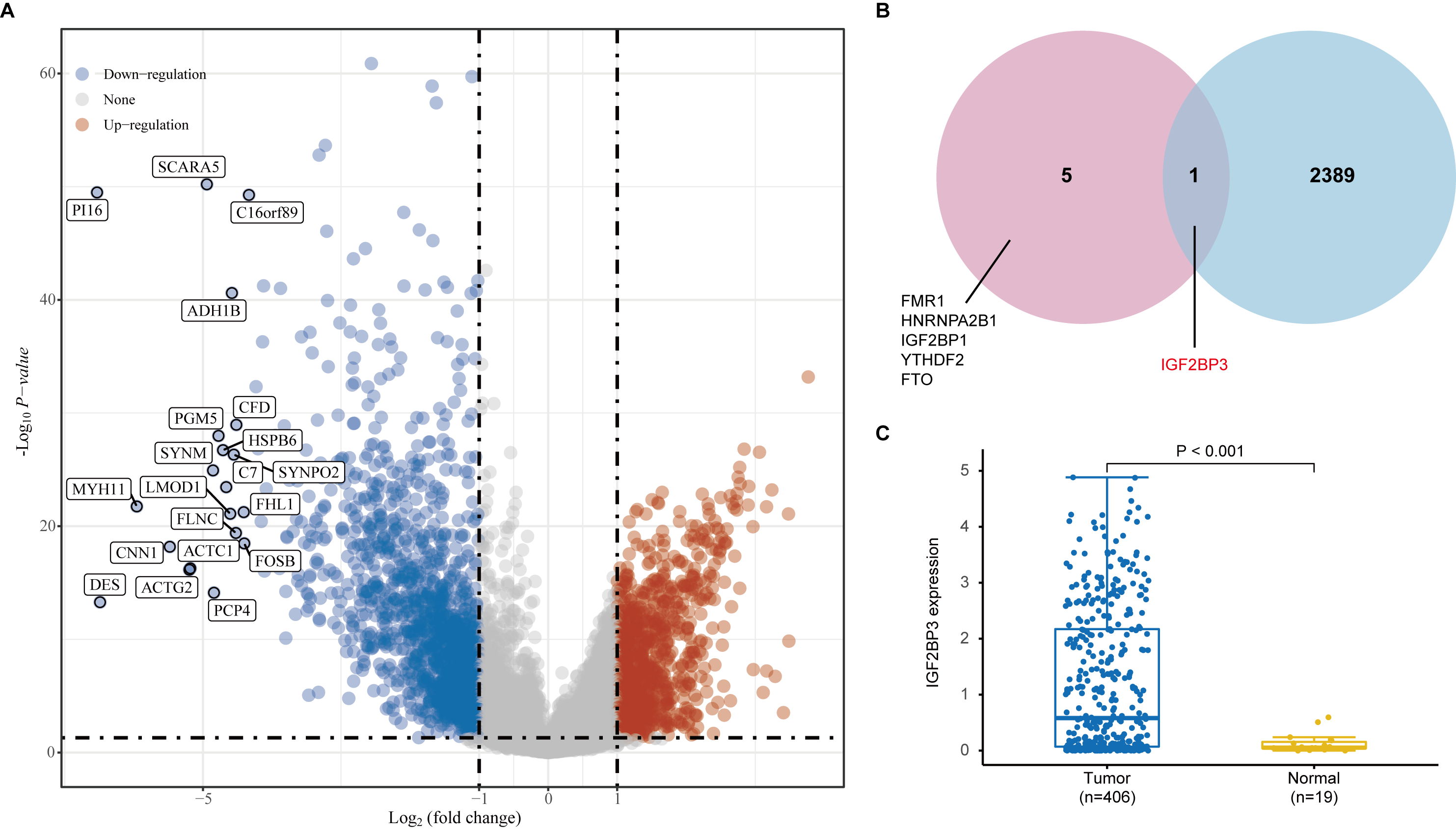
**

## Figure S3. Differentially expressed genes identified in the TCGA-BLCA cohort.

(A) Volcano plot depicting 2390 differentially expressed genes (DEGs) in the bladder tumor group, compared to the normal group in the TCGA-BLCA cohort. (B) Venn plot depicting common genes between the six treatment outcome-related m6A genes and the DEGs identified in the TCGA-BLCA cohort. (C) Expression of *IGF2BP3* in bladder tumor group and normal group in the TCGA-BLCA cohort.

**
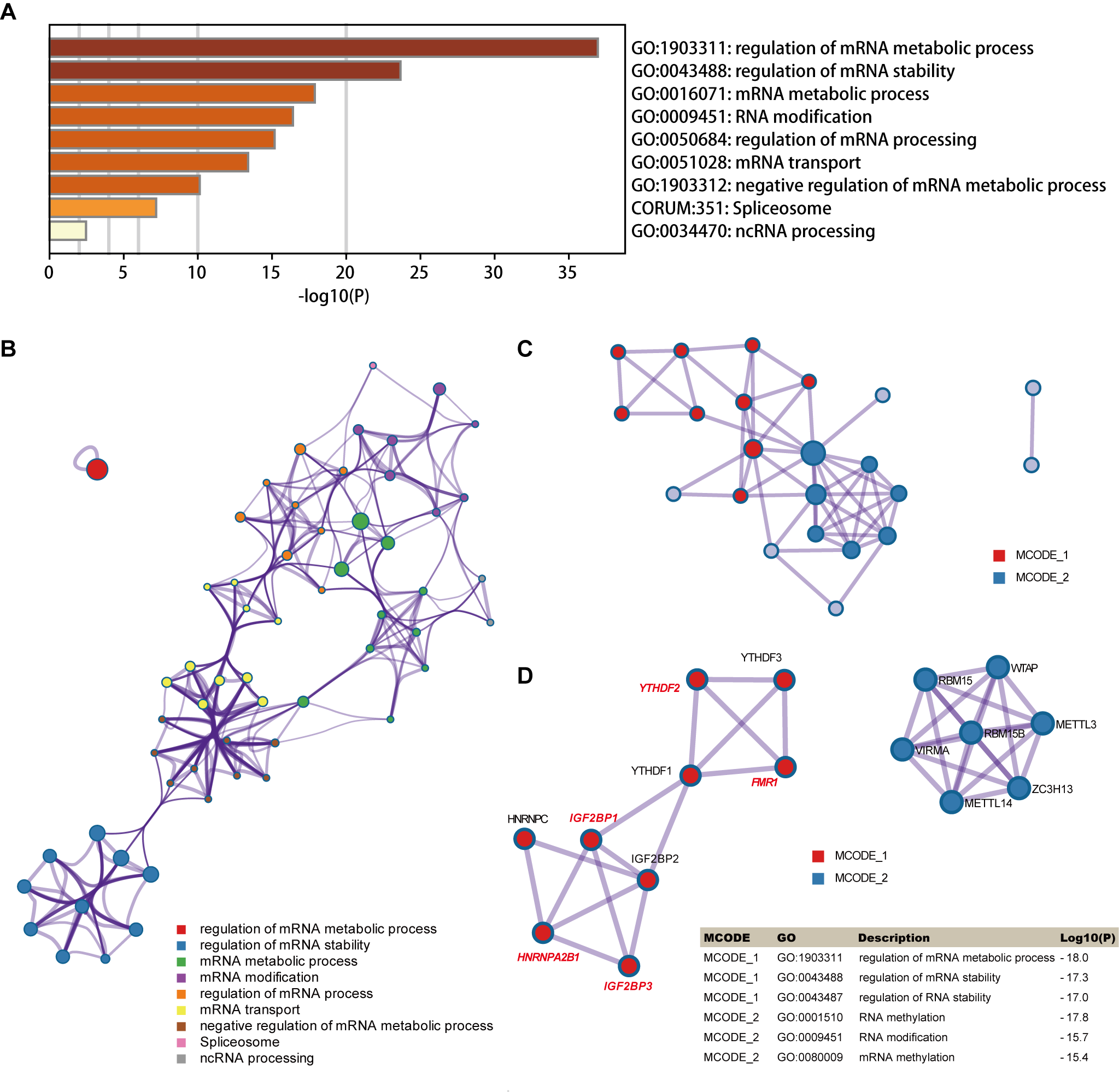
**

## Figure S4. The function network of 23 m6A related genes.

(A) Bar graph of the Gene Ontology (GO) enriched terms, colored by P-values. (B) Network of enriched terms, colored by cluster ID. (C) Protein-protein interaction network of the 23 m6A related genes. (D) Molecular Complex Detection (MCODE) components identified in the gene lists. Those treatment outcome-related m6A regulators were marked as red in the network. The three best-scoring terms by p-value have been retained as the functional description of the corresponding components, shown in the tables underneath the network plots.
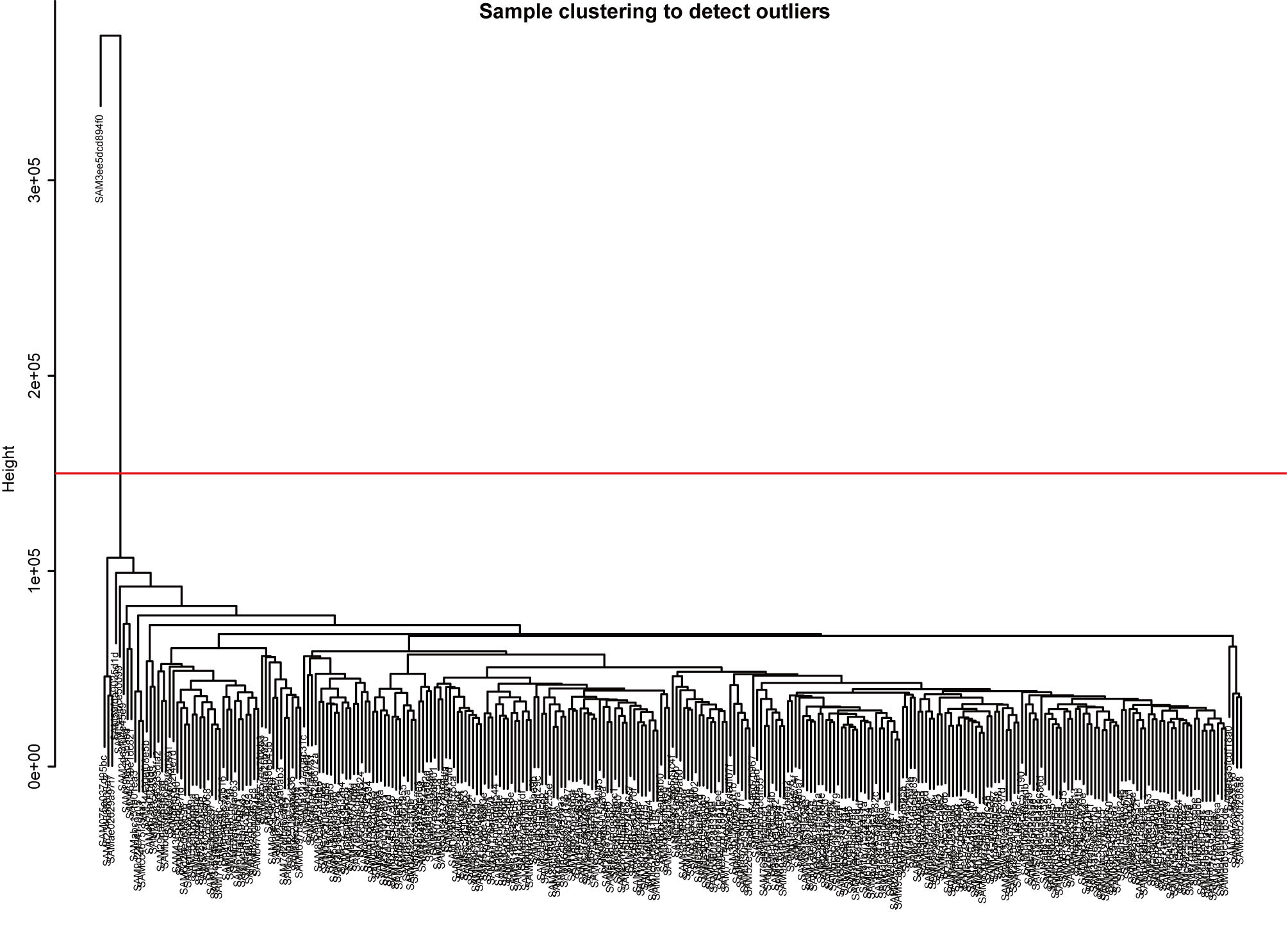


## Figure S5. Sample clustering to detect outliers.

Sample clustering was used to detect outliers. As a result, there was one outlier in the sample clustering (Sample ID: SAM3ee5dcd894f0), which was excluded from the weighted gene co-expression network analysis.

**
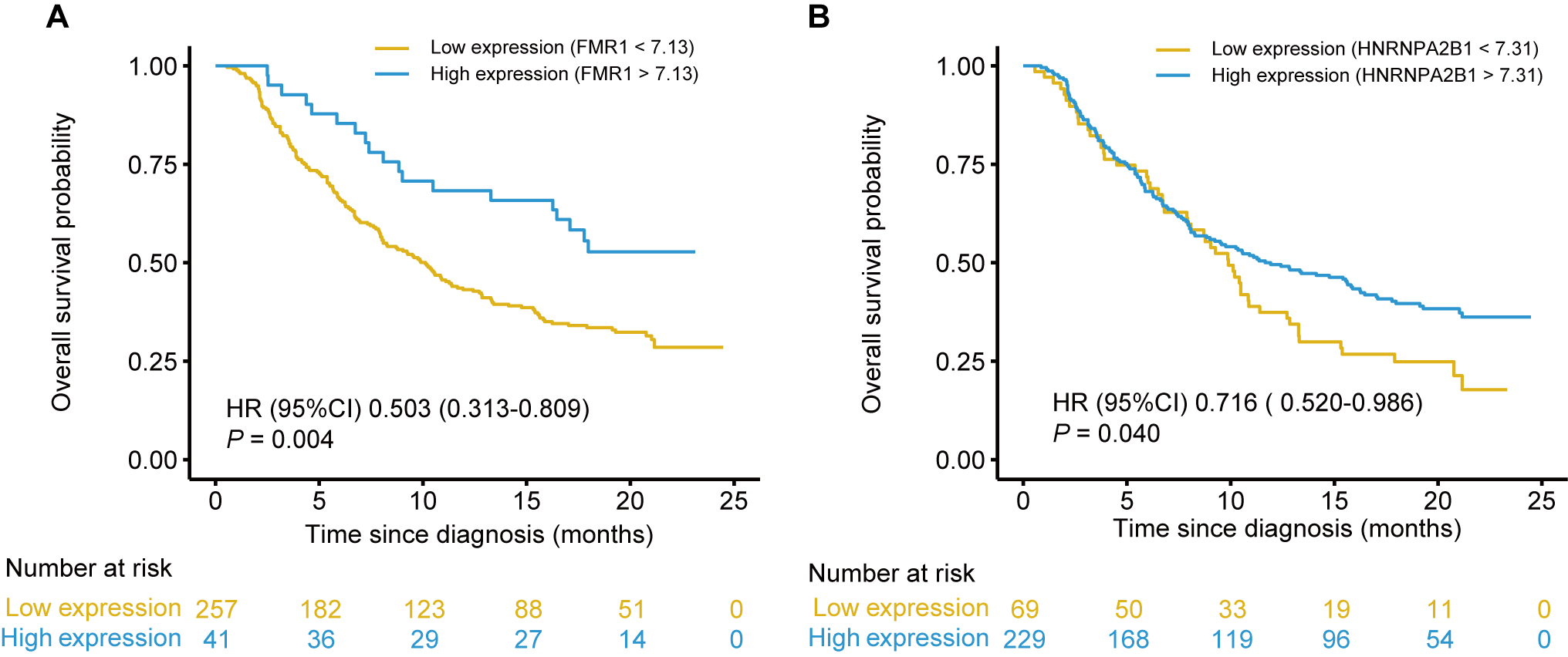
**

## Figure S6. Kaplan–Meier survival analysis for the two selected m6A related genes in the IMvigor210 cohort.

The patients were categorized into high expression group and low expression group according to the cut-off values selected via X-tile software. (A) Kaplan–Meier curves for FMR1. (B) Kaplan–Meier curves for HNRNPA2B1.

**
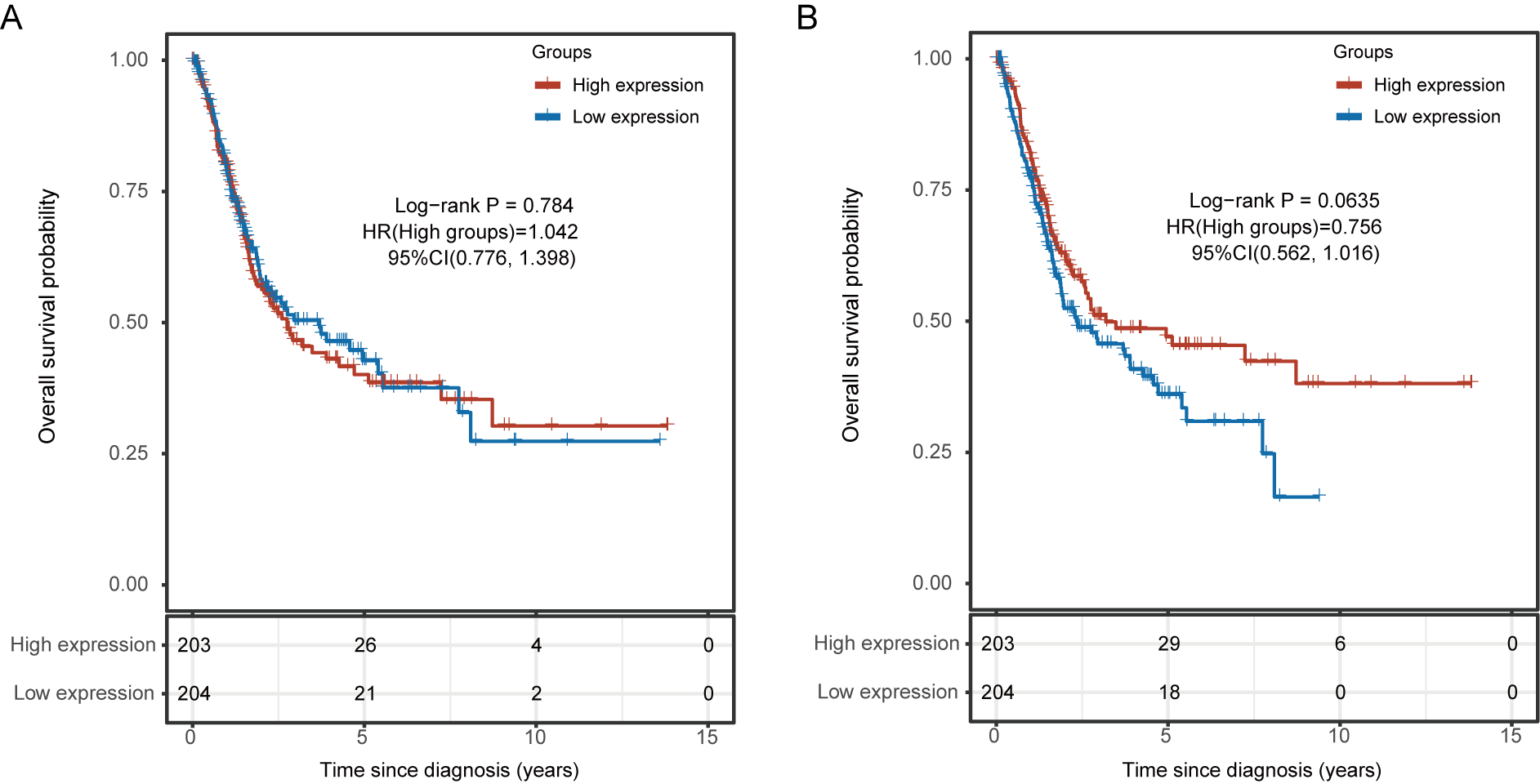
**

## Figure S7. Kaplan–Meier survival analysis for the two selected m6A related genes in the TCGA-BLCA cohort.

(A) Kaplan–Meier curves for *FMR1*. (B) Kaplan–Meier curves for *HNRNPA2B1*.
